# Quantification of DNA methylation for carcinogenic risk estimation in patients with non-alcoholic steatohepatitis

**DOI:** 10.1186/s13148-022-01379-4

**Published:** 2022-12-05

**Authors:** Junko Kuramoto, Eri Arai, Mao Fujimoto, Ying Tian, Yuriko Yamada, Takuya Yotani, Satomi Makiuchi, Noboru Tsuda, Hidenori Ojima, Moto Fukai, Yosuke Seki, Kazunori Kasama, Nobuaki Funahashi, Haruhide Udagawa, Takao Nammo, Kazuki Yasuda, Akinobu Taketomi, Tatsuya Kanto, Yae Kanai

**Affiliations:** 1 Department of Pathology, Keio University School of Medicine, 35 Shinanomachi, Shinjuku-Ku, Tokyo, 160-8582 Japan; 2 Tsukuba Research Institute, Research and Development Division, Sekisui Medical Co., Ltd., Ryugasaki, 301-0852 Japan; 3 Department of Gastroenterological Surgery I, Graduate School of Medicine, Hokkaido University, Sapporo, 060-8638 Japan; 4 Weight Loss and Metabolic Surgery Center, Yotsuya Medical Cube, Tokyo, 102-0084 Japan; 5 Department of Life Science and Technology, School of Life Science and Technology, Tokyo Institute of Technology, Yokohama, 226-8501 Japan; 6 Department of Biochemistry, Kyorin University School of Medicine, Tokyo, 181-8611 Japan; 7 Department of Metabolic Medicine and Department of Diabetes Care Medicine, Graduate School of Medicine, Osaka University, Suita, 565-0871 Japan; 8 Department of Diabetes, Endocrinology and Metabolism, Kyorin University School of Medicine, Tokyo, 181-8611 Japan; 9 Diabetes Research Center, National Center for Global Health and Medicine, Tokyo, 162-8655 Japan; 10 The Research Center for Hepatitis and Immunology, National Center for Global Health and Medicine, Ichikawa, 272-8516 Japan

**Keywords:** DNA methylation, Non-alcoholic steatohepatitis, Hepatocellular carcinoma, Carcinogenic risk estimation, Infinium assay, High-performance liquid chromatography

## Abstract

**Background:**

In recent years, non-alcoholic steatohepatitis (NASH) has become the main cause of hepatocellular carcinoma (HCC). As a means of improving the treatment of NASH-related HCCs based on early detection, this study investigated the feasibility of carcinogenic risk estimation in patients with NASH.

**Results:**

Normal liver tissue (NLT), non-cancerous liver tissue showing histological findings compatible with non-alcoholic fatty liver from patients without HCC (NAFL-O), non-cancerous liver tissue showing NASH from patients without HCC (NASH-O), non-cancerous liver tissue showing non-alcoholic fatty liver from patients with HCC (NAFL-W), non-cancerous liver tissue showing NASH from patients with HCC (NASH-W) and NASH-related HCC were analyzed. An initial cohort of 171 tissue samples and a validation cohort of 55 tissue samples were used. Genome-wide DNA methylation screening using the Infinium HumanMethylation450 BeadChip and DNA methylation quantification using high-performance liquid chromatography (HPLC) with a newly developed anion-exchange column were performed. Based on the Infinium assay, 4050 CpG sites showed alterations of DNA methylation in NASH-W samples relative to NLT samples. Such alterations at the precancerous NASH stage were inherited by or strengthened in HCC samples. Receiver operating characteristic curve analysis identified 415 CpG sites discriminating NASH-W from NLT samples with area under the curve values of more than 0.95. Among them, we focused on 21 CpG sites showing more than 85% specificity, even for discrimination of NASH-W from NASH-O samples. The DNA methylation status of these 21 CpG sites was able to predict the coincidence of HCC independently from histopathological findings such as ballooning and fibrosis stage. The methylation status of 5 candidate marker CpG sites was assessed using a HPLC-based system, and for 3 of them sufficient sensitivity and specificity were successfully validated in the validation cohort. By combining these 3 CpG sites including the *ZC3H3* gene, NAFL-W and NASH-W samples from which HCCs had already arisen were confirmed to show carcinogenic risk with 95% sensitivity in the validation cohort.

**Conclusions:**

After a further prospective validation study using a larger cohort, carcinogenic risk estimation in liver biopsy specimens of patients with NASH may become clinically applicable using this HPLC-based system for quantification of DNA methylation.

**Supplementary Information:**

The online version contains supplementary material available at 10.1186/s13148-022-01379-4.

## Background

Non-alcoholic steatohepatitis (NASH), a hepatic manifestation of metabolic syndrome resulting in the development of liver cirrhosis [1], has shown an alarming increase in recent years [2, 3]. Although hepatitis B virus (HBV) or hepatitis C virus (HCV) infection followed by chronic hepatitis and liver cirrhosis used to be the main cause of hepatocellular carcinoma (HCC), there is evidence that NASH is becoming another precancerous condition for HCC [4, 5]. It is well known that not only genomic but also epigenomic alterations participate in carcinogenesis in various human organs [6, 7]. DNA methylation alterations are one of the most important epigenomic changes resulting in chromosomal instability and aberrant expression of tumor-related genes [8–10]. In fact, our previous genome-wide DNA methylation analysis using the Infinium assay with pathological tissue specimens has revealed that NASH-specific DNA methylation alterations, which differ from such alterations occurring during viral hepatitis, are inherited by or strengthened in NASH-related HCCs [11]. DNA methylation alterations occurring under the conditions of NASH participated in NASH-related multistage hepatocarcinogenesis through aberrant expression of specific tumor-related genes, such as *WHSC1* [11], *TRIM4, PRC1* and *TUBA1B* [12].

On the other hand, carcinogenic risk estimation is a powerful tool for preemptive medicine [13–15]. Although intensive carcinogenesis surveillance at the precancerous stage is desirable, since HCC resulting from NASH-related cirrhosis has been reported 11% [16], it may not be feasible in all NASH patients. If it were possible to predict the risk of NASH-related hepatocarcinogenesis using liver biopsy specimens, NASH patients at high risk might be motivated to visit hospitals more frequently, allowing HCCs to be detected early by close follow-up. Since DNA methylation alterations at the precancerous stage are stably maintained on the DNA double strand by covalent bonding through a maintenance methylation mechanism involving DNA methyltransferase 1 (DNMT1) [17, 18], we have successfully established carcinogenic risk estimation criteria for viral hepatitis-related HCC [19, 20] and urothelial carcinoma [21, 22] based on DNA methylation profiles. By analogy with risk estimation for such carcinomas, we have been investigating the feasibility of carcinogenic risk estimation for NASH patients based on quantification of DNA methylation.

For carcinogenic risk estimation based on this approach, it is crucial to develop an analytical system capable of delivering data readily, quickly and precisely, especially for clinical samples that include a variety of cell lineages. To devise such a system that satisfies these requirements, we have developed an anion-exchange high-performance liquid chromatography (HPLC) column with electrostatic and hydrophobic properties for detection of methylated DNA [23]. Our previous study had indicated that HPLC analysis using this newly developed anion-exchange column is useful for DNA methylation diagnostics using pathological tissue samples [23–25].

In the present study, designed to make carcinogenic risk estimation possible for patients with NASH, genome-wide screening using the Infinium assay and DNA methylation quantification using our newly developed HPLC-based system were performed using 171 liver tissue specimens in an initial cohort and 55 in a validation cohort (226 samples in total).

## Methods

### Patients and tissue samples

As an initial cohort, we used 22 paired samples of non-cancerous liver tissue (N) and corresponding cancerous tissue (T) obtained by partial hepatectomy from 22 HCC patients whose *N* samples showed histological features compatible with NASH. All 22 patients were negative for both HBV surface antigen and anti-HCV antibody. NASH stage was evaluated microscopically on the basis of the histological scoring system for NASH [26] and the Brunt classification [27]. The HCCs were diagnosed histologically in accordance with the World Health Organization classification [28] and the tumor–node–metastasis classification [29]. For comparison, 36 samples of normal control liver tissue (NLT), obtained by partial hepatectomy from 36 patients with liver metastases of primary colorectal cancers without HBV or HCV infection, chronic hepatitis, liver cirrhosis or HCC, were examined. These patients did not receive any preoperative treatment and underwent partial hepatectomy at the National Cancer Center Hospital.

Liver biopsy specimens showing histological features compatible with NASH were obtained from 91 morbidly obese patients during bariatric surgery under general anesthesia at the Yotsuya Medical Cube. Before inclusion in this study, these patients had been informed of the risk of obesity-associated NASH [30], the therapeutic benefit of early NASH diagnosis and the invasiveness of the liver biopsy procedure. None of these 91 patients had HBV or HCV infection, or HCC. The 22 N samples obtained by partial hepatectomy from patients with HCC and the 91 N samples from biopsy specimens of patients with morbid obesity but without HCC are abbreviated hereafter as NASH-W and NASH-O, respectively. On this basis, 36 NLT samples, 91 NASH-O samples, 22 NASH-W samples and 22 T samples (171 samples in total) comprised the initial cohort (Table 1).

**Table 1:** Liver tissue samples in the initial and validation cohorts

Cohorts	Samples	Number of specimens	Histological findings in non-cancerous liver tissue (N)	Coexistence of hepatocellular carcinoma (HCC)	Hepatitis virus infection
Initial cohort	Normal liver tissue (**NLT**) from patients with liver metastasis of colorectal cancer (CRC)	36	Normal	No	No
	N showing non-alcoholic steatohepatitis (NASH) from patients with morbid obesity with**o**ut HCC (**NASH-O**)	91	NASH	No	No
	N showing NASH from patients **w**ith HCC (**NASH-W**)	22	NASH	Yes	No
	HCC (**T**)	22	NASH	N/A	No
Validation cohort	**NLT** from patients with liver metastasis of CRC	22	Normal	No	No
	N showing non-alcoholic fatty liver (NAFL), but not satisfying the criteria for NASH, from patients with liver metastasis of CRC (**NAFL-O**)	9	NAFL	No	No
	N showing NASH from patients with liver metastasis of CRC (**NASH-O**)	3	NASH	No	No
	N showing NAFL from patients with HCC (**NAFL-W**)	10	NAFL	Yes	No
	**NASH-W**	11	NASH	Yes	No

As a validation cohort, 11 NASH-W samples were obtained by partial hepatectomy from 11 HCC patients. In addition, 10 N samples showing histological features compatible with non-alcoholic fatty liver (NAFL) were obtained by partial hepatectomy from 10 HCC patients (abbreviated hereafter as NAFL-W). Although NAFL is a pathological condition showing less severe necroinflammation and not satisfying the histological criteria for NASH, it is categorized as non-alcoholic fatty liver disease (NAFLD) along with NASH [31]. For comparison, 22 NLT samples, 9 N samples showing histological features compatible with NAFL (NAFL-O samples) and 3 N samples showing histological features compatible with NASH (NASH-O samples), were obtained by partial hepatectomy from 34 patients with liver metastases of primary colorectal cancers without HBV or HCV infection or HCC. These patients did not receive any preoperative treatment and underwent partial hepatectomy at Hokkaido University Hospital. Therefore, 22 NLT samples, 9 NAFL-O samples, 3 NASH-O samples, 10 NAFL-W samples and 11 NASH-W samples (55 samples in total) comprised the validation cohort (Table 1).

The age, sex and clinicopathological backgrounds of the patients from whom the 171 and 55 samples in the initial and validation cohorts, respectively, were obtained, are summarized in Additional file 1: Table S1. After surgical removal or biopsy, tissue specimens were immediately frozen and stored until use for research in accordance with the Japanese Society of Pathology Guidelines for the handling of pathological tissue samples for genomic research [32]. This study was approved by the Ethics Committees of the National Cancer Center, the National Center for Global Health and Medicine, Yotsuya Medical Cube and Hokkaido University Hospital, and was performed in accordance with the Declaration of Helsinki. All patients gave written informed consent for inclusion of their specimens in this study.

### Genome-wide DNA methylation screening by Infinium assay

High-molecular-weight DNA from fresh frozen tissue samples was extracted using either a QIAamp DNA Micro kit (Qiagen, Valencia, CA), or phenol–chloroform, followed by dialysis. Five-hundred-nanogram aliquots of DNA were subjected to bisulfite conversion using an EZ DNA Methylation-Gold™ Kit (Zymo Research, Irvine, CA, USA). DNA methylation status at 485,764 CpG loci was examined at single-CpG resolution using the Infinium HumanMethylation450 BeadChip (Illumina, San Diego, CA, USA) [33]. After hybridization, the specifically hybridized DNA was fluorescence-labeled by a single-base extension reaction and detected using an iScan reader (Illumina) in accordance with the manufacturer’s protocol.

The data were then assembled using GenomeStudio methylation software (Illumina). At each CpG site, the ratio of the fluorescence signal was measured using a methylated probe relative to the sum of the methylated and unmethylated probes, i.e., the so-called *β*-value, which ranges from 0.00 to 1.00, representing the values for a fully unmethylated and fully methylated individual CpG site, respectively. Infinium data for a proportion of the initial cohort were also included in our previous paper focusing on differences in DNA methylation profiles between NASH-related HCC and viral hepatitis-related HCC [11] and deposited in the GEO database (https://www.ncbi.nlm.nih.gov/geo/, Accession number GSE89852). In addition, Infinium data newly acquired in the present study are deposited in the GEO database (Accession number GSE183468).

### Anion-exchange high-performance liquid chromatography (HPLC) analysis

We have developed a novel anion-exchange column for HPLC with electrostatic and hydrophobic properties. Both cytosine and thymine, corresponding to methylated and unmethylated cytosine after bisulfite modification, respectively, are captured by electrostatic interaction and then discriminated from each other by their hydrophobic interactions in our column [23]. As described previously, our column packing material was prepared using a three-step procedure [23]. Briefly, a reactor equipped with a stirrer was filled with 2000 mL of a 3% w/w aqueous solution of polyvinyl alcohol, and a mixture of 200 g tetraethylene glycol dimethacrylate, 100 g triethylene glycol dimethacrylate, 100 g glycidyl methacrylate and 1 g benzoyl peroxide was added to the solution. The contents were heated to 80 °C with stirring and polymerized at that temperature for 1 h in a nitrogen atmosphere. In this initial step, polymer particles with hydrophobic properties were formed.

Subsequently, a monomer solution of 100 g ethyl methacrylate trimethyl ammonium chloride dissolved in 100 mL ion-exchange water was added to the reactor, and the contents were polymerized at 80 °C for 2 h in a nitrogen atmosphere to prepare a polymerization composite. This composite was rinsed with ion-exchange water and then with acetone. In this second step, polymer chains with quaternary ammonium groups were introduced onto the surface of the polymer particles.

Finally, 10 g of polymer particles were dispersed in 100 mL ion-exchange water to prepare an unreacted slurry. Next, 10 mL N,N-dimethylamino propyl amine was added to the slurry with stirring, and the contents were allowed to react at 70 °C for 4 h. After completion of the reaction, the supernatant was removed using a centrifugal separator, Himac CR20G (Hitachi Koki Co., Ltd., Tokyo, Japan), and the remaining contents were rinsed with ion-exchange water. After rinsing, any additional supernatant was removed using a centrifugal separator. Rinsing with ion-exchange water was then performed an additional 4 times. In this final step, tertiary amino groups were introduced onto the surface of the polymer particles.

Bisulfite-converted genomic DNA in each sample and fully unmethylated and fully methylated control DNA (Qiagen) were amplified by PCR encompassing the candidate marker CpG sites using primers described in Additional file 1: Table S2. The PCR products were then subjected to HPLC analysis on an LC-20A system (Shimadzu Corporation, Kyoto, Japan) equipped with a stainless steel column (150 × 4.6 mm I.D.) filled with our original packing material. Eluent buffer A was 25 mmol/L MES-NaOH (pH 6.0), and Eluent buffer B was the same buffer containing 2 mol/L guanidine sulfate. The PCR products were separated on a gradient of 30–50% buffer B for 10 min at a flow rate of 1.0 mL/min. The separated PCR products were detected at 260 nm. HPLC analysis was performed at a column temperature of 70 °C.

### Statistical analysis

In the Infinium assay, the call proportions (*P* value < 0.01 for detection of signals above the background) for 751 probes in all of the tissue samples in the initial cohort (36 NLT samples, 91 NASH-O samples, 22 NASH-W and 22 NASH-T samples) were less than 90%. Since such a low proportion may have been attributable to polymorphism at the probe CpG sites, these 751 probes were excluded from the present assay, as described previously [34, 35]. In addition, all 11,648 probes on chromosomes X and Y were excluded to avoid any gender-specific methylation bias, leaving a final total of 473,365 autosomal CpG sites.

Infinium probes showing significant differences in DNA methylation levels between NLT samples and NASH-W samples were defined by Welch’s *t* test and adjusted by Bonferroni correction. Alterations of DNA methylation levels from NLT to NASH-W and then T were examined by the Jonckheere–Terpstra trend test. The receiver operating characteristic (ROC) curve was generated using *β* values based on the Infinium assay and the relative DNA methylation rates obtained by HPLC analysis for discrimination of the NASH-W samples from NLT samples in the initial cohort. The Youden index [36] was used as the cutoff value for such discrimination. Multivariate analysis was performed to predict the coincidence of HCC based on the DNA methylation profiles. Differences at *P* values of less than 0.05 were considered statistically significant. All statistical analyses were performed using the statistical program RStudio (RStudio Inc., Boston, MA, https://www.rstudio.com) and the R software package (R Foundation for Statistical Computing, https://www.r-project.org).

## Results

### Discrimination of NASH-W samples from NLT samples based on DNA methylation profiles in the initial cohort

**Fig. 1:**
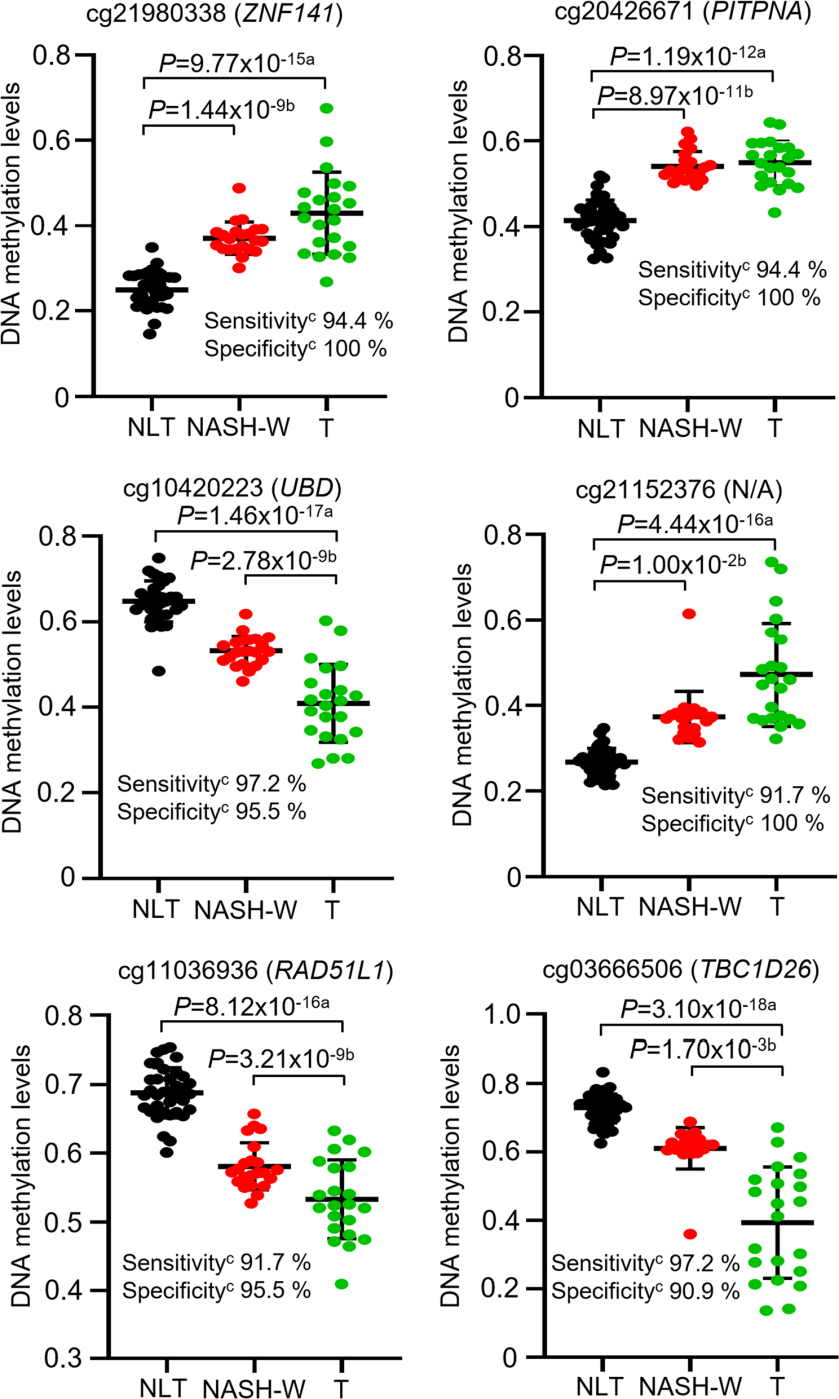
Scattergrams of DNA methylation levels obtained by the Infinium assay for representative CpG sites showing area under the curve values of more than 0.95 in receiver operating characteristic curve analysis for discrimination between non-cancerous liver tissue showing non-alcoholic steatohepatitis (NASH) derived from partial hepatectomy specimens from patients with hepatocellular carcinoma (HCC) (NASH-W) (*n* = 22, red) and normal liver tissue (NLT) samples (*n* = 36, black). The Infinium probe ID and gene symbol are shown at the top of each panel. N/A, not annotated (designed in the intergenic regions). DNA methylation alteration at the NASH stage compared to NLT was inherited by or strengthened in NASH-related HCC (T) samples (*n* = 22, green). Using each CpG site and its cutoff value described in Additional file 1: Table S3, NASH-W samples were discriminated from NLT samples with sufficient sensitivity and specificity (90.9–100%). ^a^*P* values by Welch’s *t* test. ^b^*P* values by Jonckheere–Terpstra trend test. ^c^Sensitivity was defined as the ratio of the number of tissue samples diagnosed as NASH-W based on the criteria described in Additional file 1: Table S3 relative to the exact number of NASH-W samples, and specificity was defined as the ratio of the number of tissue samples not diagnosed as NASH-W based on the criteria relative to the exact number of NLT samples

Four thousand and fifty CpG sites showed significant differences in DNA methylation levels between the 22 NASH-W samples and the 36 NLT samples in the initial cohort (Welch’s *t* test, adjusted by Bonferroni correction [*α* = 1.12 × 10^−7^] and a Δ*β*_NASH-W-NLT_ value of more than 0.1 or less than − 0.1), indicating that DNA methylation alterations had occurred in the NASH-W samples, which showed histological features compatible with NASH and from which HCC had arisen, in comparison with NLT samples. A Venn diagram showing the number of common and individual differentially methylated probes among the NLT, NASH-O, NASH-W and T samples is shown in Additional file 1: Fig. S1. On 3234 of the 4050 probes, DNA methylation alterations in NASH-W samples relative to NLT samples were inherited by or strengthened in the T samples themselves (Jonckheere–Terpstra trend test) (Fig. 1). ROC curves were then constructed for the 3234 CpG sites for discrimination of NASH-W samples (origin of the NASH-related HCC) from NLT samples.

Among the 3234 CpG sites, 415 showed AUC values larger than 0.95; 209 and 206 CpG sites showed DNA hyper-(*β*_NASH-W_ > *β*_NLT_) and hypo-(*β*_NASH-W_ < *β*_NLT_) methylation in NASH-W samples relative to NLT samples, respectively. The Youden index was used as a cutoff value for each of the 415 CpG sites to discriminate NASH-W samples from NLT samples. The sensitivity and specificity using each of the 415 CpG sites for such discrimination are shown in Additional file 1: Table S3. As shown by the representative scattergrams in Fig. 1, NASH-W samples were discriminated from NLT samples based on the DNA methylation levels of each candidate marker CpG site with sufficient sensitivity (91.7–97.2% in Fig. 1) and specificity (90.9–100% in Fig. 1).

Pathway analysis was performed using the 415 probes that showed AUC values of more than 0.95 for discrimination of NASH-W samples from NLT samples and were designed for the 339 genes by Reactome analysis (https://reactome.org). Seventy pathway maps in which such probes were significantly (*P* < 0.05) accumulated are summarized in Additional file 1: Table S4. The top pathways frequently participate in chromatin modification, autophagy, cell cycle regulation and DNA repair (i.e., R-HSA-3247509 (*P* = 0.000904), R-HSA-1632852 (*P* = 0.001041), R-HSA-68886 (*P* = 0.001629) and R-HSA-5693565 (*P* = 0.006073), respectively) (see Additional file 1: Table S4).

### Discrimination of NASH-W samples from NASH-O samples based on DNA methylation profiles in the initial cohort

For carcinogenic risk estimation during follow-up for patients with NASH, the DNA methylation status of NASH-W samples from which NASH-related HCCs have already arisen should be compared with that of NASH-O samples from patients who have not developed HCCs. Therefore, for discrimination of the 22 NASH-W samples from the 91 NASH-O samples in the initial cohort, sensitivity and specificity were calculated using the cutoff values shown in Additional file 1: Table S3. Since the incidence of HCC resulting from NASH-related cirrhosis is reportedly 11%, it is feasible that some patients from whom NASH-O samples have been obtained will develop HCC in the future if appropriate intervention is not provided. Among the 415 CpG sites, 21 showed 85% or more specificity for discrimination of NASH-W samples from NASH-O samples: These 21 CpG sites predicted that up to 15% of NASH-O samples would have the same carcinogenic risk as NASH-W samples. Since it makes clinical sense to closely follow up the top 15% of patients at high risk of developing NASH-related HCC, these 21 CpG sites are summarized in Table 2 as candidate marker CpG sites for carcinogenic risk estimation.

**Table 2:** The 21 CpG sites for which receiver operating characteristic curve analysis showed area under the curve (AUC) values larger than 0.95 for discrimination of non-cancerous liver tissue samples showing non-alcoholic steatohepatitis (NASH) from partial hepatectomy specimens from patients with hepatocellular carcinoma (HCC) (NASH-W) from normal liver tissue samples (NLT), and larger than 85% specificity for discrimination of NASH-W samples from non-cancerous liver tissue samples showing NASH from biopsy specimens from patients with morbid obesity but without HCC (NASH-O)

Probe ID^a^	Gene symbol^b^	Gene region^c^	CpG type^d^	Discrimination of NASH-W from NLT		Discrimination of NASH-W from NASH-O
				AUC	DNA methylation status^e^	Specificity^f^
cg00431813	*C7orf50*	Gene body	S_shelf/N_shore	0.960	NASH-W > NLT	0.978
cg05393736	*NRBF2*	TSS1500	N_shore	0.960	NASH-W > NLT	0.956
cg09580822	N/A			0.974	NASH-W < NLT	0.945
cg11820154	N/A			0.953	NASH-W > NLT	0.945
cg14950303	*FOXD4L1*	TSS1500	N_shore/N_shelf	0.957	NASH-W > NLT	0.912
cg15050398	*LOC401242*	Gene body	N_shelf	0.958	NASH-W < NLT	0.912
cg23785719	*STK17A*	TSS200	N_shore	0.962	NASH-W > NLT	0.912
cg26583598	*ZSWIM6*	Gene body		0.953	NASH-W > NLT	0.901
cg18210511	*ZC3H3*	Gene body	Island	0.956	NASH-W > NLT	0.901
cg05861743	*APOM*	Gene body	N_shelf/S_shelf	0.951	NASH-W > NLT	0.890
cg14288091	*NIPAL2*	Gene body/1st intron	N_shore	0.952	NASH-W < NLT	0.890
cg26385097	N/A			0.953	NASH-W < NLT	0.890
cg22083325	*LOC440461*	TSS200	N_shelf/Island	0.982	NASH-W > NLT	0.889
cg09580859	*ZC3H3*	Gene body	Island	0.951	NASH-W > NLT	0.879
cg13719443	*LOC285847*	Gene body	S_shore	0.952	NASH-W < NLT	0.879
cg26894854	*SLC45A3*	Gene body	N_shore	0.968	NASH-W > NLT	0.879
cg04662828	N/A			0.963	NASH-W < NLT	0.868
cg11152384	N/A			0.957	NASH-W < NLT	0.867
cg08912801	N/A			0.974	NASH-W < NLT	0.857
cg16319310	N/A			0.987	NASH-W < NLT	0.857
cg19861632	*GPR125*	Gene body		0.963	NASH-W > NLT	0.857

^a^Probe ID of the Infinium HumanMethylation450 BeadChip

^b^National Center for Biotechnology Information (NCBI) database (Genome Build 37)

^c^TSS1500 (from 200 bp upstream of the transcription start site [TSS] to 1500 bp upstream of it), TSS200 (from TSS to 200 bp upstream of it), 1st intron and gene body (2nd exon and downstream) were identified based on the RefSeq database (http://www.ncbi.nlm.nih.gov/refseq/)

^d^CpG islands, island shores (2000-bp regions adjacent to a CpG island) and island shelves (2000-bp regions adjacent to an island shore) were identified based on the University of California, Santa Cruz (UCSC) genome browser (https://genome.ucsc.edu/)

^e^NASH-W < NLT: the sample was predicted to be NASH-W and/or carcinogenic risk-positive when its DNA methylation level was lower than the cutoff value; NASH-W > NLT: the sample was predicted to be NASH-W and/or carcinogenic risk-positive when its DNA methylation level was higher than the cutoff value

^f^Specificity was defined as the ratio of the number of tissue samples not diagnosed as NASH-W based on the criteria relative to the exact number of NASH-O samples. N/A, not annotated (designed in the intergenic regions)

The correlation between DNA methylation levels of the 13 genes, which are included in Table 2 and for which expression data are available in The Cancer Genome Atlas (TCGA) database (https://www.cancer.gov/about-nci/organization/ccg/research/structural-genomics/tcga), was examined using 384 cancerous and non-cancerous liver tissues deposited in the TCGA database (Additional file 1: Table S5). Only three of 13 genes showed a significant correlation between the levels of DNA methylation and expression (*P* < 0.05 and *r* < -0.2 or *r* > 0.2), indicating that even DNA methylation alterations at CpG sites not involved in the regulation of expression would be potentially excellent surrogate markers.

On the other hand, because of concerns about the effects of metastasis of colorectal cancer on the DNA methylation levels of the NLT samples, we compared the DNA methylation levels of the 21 CpG sites included in Table 2 between data for normal liver tissue from 40 healthy individuals (GSE107038) deposited in the GEO database (https://www.ncbi.nlm.nih.gov/geo/query/acc.cgi?acc=GSE107038) and our own data for NASH-W samples. Even when compared to data for normal healthy liver tissue in the database, significant differences were successfully validated (Additional file 1: Table S6), excluding the possibility that the differences between NLT and NASH-W were attributable to the effects of liver metastasis from colorectal cancer.

Multivariate analysis was then performed using the 91 NASH-O samples and the 22 NASH-W samples in the initial cohort (113 NASH samples in total). The DNA methylation levels at all 21 CpG sites discriminated NASH-W samples from NASH-O samples independently from histopathological findings, such as ballooning and fibrosis stage (Brunt classification), which are reportedly correlated with pathological progression [26, 37] (see Additional file 1: Table S7). Among the 21 CpG sites, the top 10 showing the highest odds ratio (141.119 to 1138.492) of being NASH-W samples, i.e., coincidence of HCC, are summarized in Table 3.

**Table 3:** Multivariate analysis using the 91 samples of non-cancerous liver tissue showing non-alcoholic steatohepatitis (NASH) derived from biopsy specimens of patients with morbid obesity but without hepatocellular carcinoma (HCC) (NASH-O samples) and the 22 samples of non-cancerous liver tissue showing NASH derived from partial hepatectomy specimens of patients with HCC (NASH-W samples) in the initial cohorts

Probe ID^a^	Variables	Odds ratio^b^	95% confidence interval	*P* value^c^
cg11820154	Ballooning^d^	0.460	0.031–6.765	0.571
	Brunt classification^e^	0.347	0.100–1.207	0.096
	DNA methylation quantification	1138.492	52.740–24,576.521	7.13 × 10^−6^
cg26583598	Ballooning^d^	0.692	0.051–9.460	0.782
	Brunt classification^e^	0.286	0.090–0.915	0.035
	DNA methylation quantification	1035.707	54.365–19,731.273	3.89 × 10^−6^
cg18210511	Ballooning^d^	8.990	0.219–369.308	0.247
	Brunt classification^e^	0.482	0.207–1.123	0.091
	DNA methylation quantification	248.725	30.517–2027.198	2.56 × 10^−7^
cg00431813	Ballooning^d^	5.786	0.132–252.827	0.362
	Brunt classification^e^	0.686	0.275–1.714	0.420
	DNA methylation quantification	232.059	31.166–1727.915	1.05 × 10^−7^
cg19861632	Ballooning^d^	2.047	0.191–21.929	0.554
	Brunt classification^e^	0.610	0.302–1.232	0.168
	DNA methylation quantification	207.072	20.883–2053.237	5.20 × 10^−6^
cg05861743	Ballooning^d^	1.491	0.133–16.687	0.746
	Brunt classification^e^	0.503	0.229–1.106	0.088
	DNA methylation quantification	190.885	24.574–1482.750	5.14 × 10^−7^
cg09580822	Ballooning^d^	1.491	0.124–15.383	0.795
	Brunt classification^e^	0.503	0.248–1.235	0.148
	DNA methylation quantification	190.885	23.957–1361.603	4.61 × 10^−7^
cg09580859	Ballooning^d^	9.892	0.299–327.815	0.200
	Brunt classification^e^	0.506	0.226–1.133	0.098
	DNA methylation quantification	180.393	24.567–1324.612	3.27 × 10^−7^
cg14950303	Ballooning^d^	1.256	0.114–13.824	0.853
	Brunt classification^e^	0.702	0.315–1.565	0.387
	DNA methylation quantification	144.050	21.556–962.621	2.92 × 10^−7^
cg26385097	Ballooning^d^	1.577	0.145–17.140	0.708
	Brunt classification^e^	0.595	0.275–1.288	0.188
	DNA methylation quantification	141.119	20.394–976.513	5.30 × 10^−7^

^a^Probe ID of the Infinium HumanMethylation450 BeadChip

^b^Top 10 CpG sites showing the highest odds ratio of being NASH-W samples are summarized in this table. The results of multivariate analysis for all 21 CpG sites are shown in Additional file 1: Table S7

^c^*P* values less than 0.05 are underlined

^d^Ballooning was evaluated according to the non-alcoholic fatty disease activity score (Ref. 26)

^e^Fibrosis stage was defined according to the Brunt classification (Ref. 27)

### Carcinogenic risk estimation by anion-exchange HPLC analysis

Since our anion-exchange HPLC system using the newly developed column is capable of delivering data readily, quickly and precisely for DNA methylation diagnostics [23], we examined its potential clinical application for carcinogenic risk estimation in patients with NASH. PCR products encompassing the 21 marker CpG sites generated from fully unmethylated and fully methylated control DNA were applied to the anion-exchange HPLC system. Among these 21 sites, the separation of 0% and 100% control DNA fragments encompassing 5 CpG sites (cg09580822, cg15050398, cg18210511, cg09580859 and cg13719443, Additional file 1: Table S2) was excellent on chromatograms. Therefore, we used these 5 marker CpG sites for further HPLC-based carcinogenic risk estimation.

**Fig. 2:**
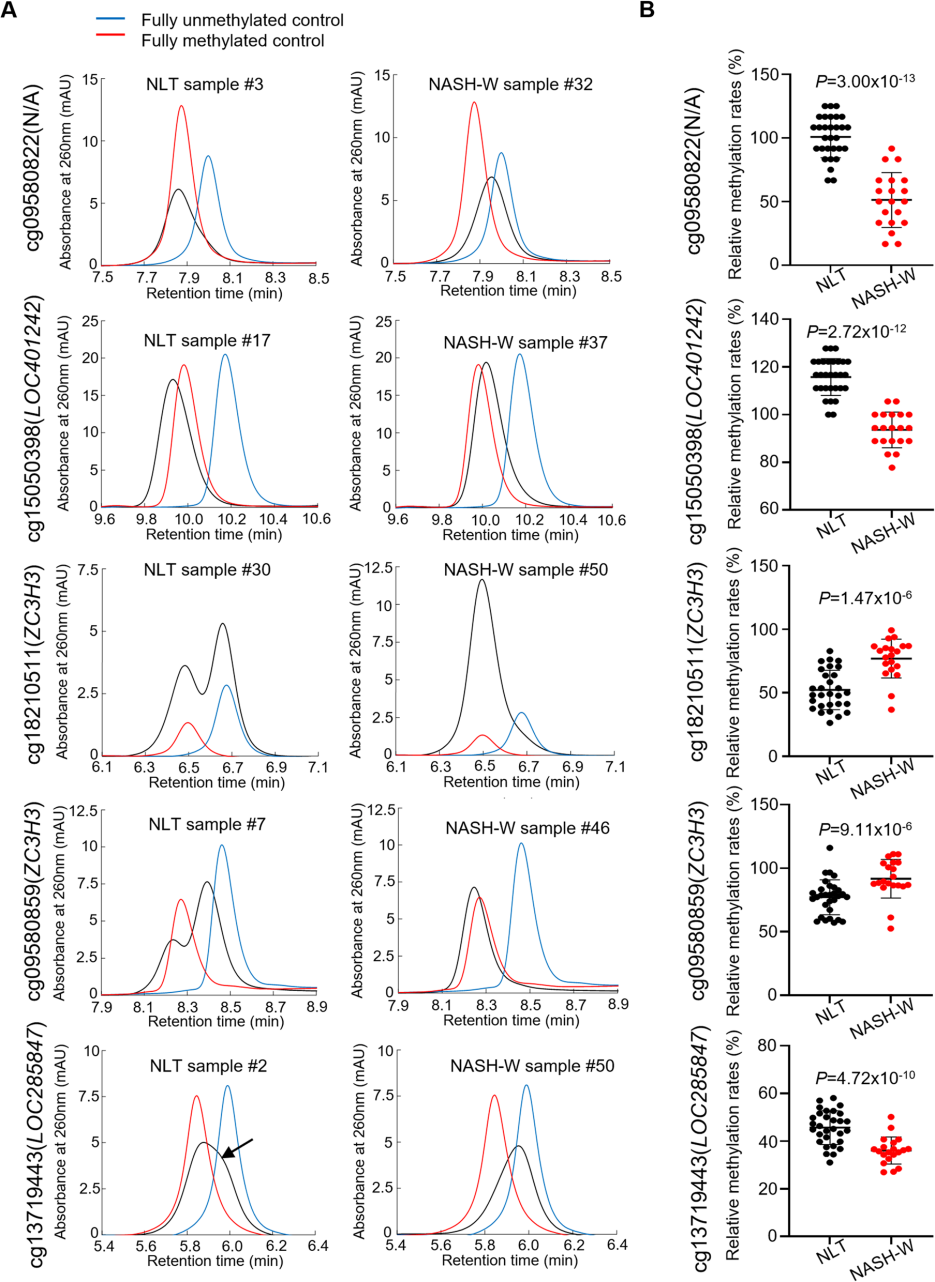
High-performance liquid chromatography (HPLC) analysis using the newly developed anion-exchange column for specimens of normal liver tissue (NLT) (*n* = 30) and non-cancerous liver tissue showing non-alcoholic steatohepatitis (NASH) derived from partial hepatectomy specimens from patients with hepatocellular carcinoma (HCC) (NASH-W) (*n* = 20) in the initial cohort. **A** Chromatograms of PCR products encompassing the marker CpG sites (cg09580822, g15050398, cg18210511, cg09580859 and cg13719443) for representative tissue samples (black). Gene names are shown on the left side. N/A: not annotated (the probe was located within the intergenic region). Chromatograms of fully unmethylated and fully methylated control DNAs are shown in blue and red, respectively. For cg09580822 and cg15050398, chromatograms with a “single peak” were obtained in both NLT and NASH-W. For cg18210511 and cg09580859, NLT showed a “bimodal peak” pattern, whereas a “single peak” pattern was observed in NASH-W. For cg15050398, NLT showed a “single peak with a shoulder” (indicated by an arrow) pattern. **B** Scattergrams of the relative DNA methylation rates obtained by HPLC analysis for cg09580822, g15050398, cg18210511, cg09580859 and cg13719443. Such rates are described in “Results” section and differed significantly between NLT (black) and NASH-W (red) samples. *P* values were obtained by Welch’s *t* test

Thirty NLT samples and 20 NASH-W samples in the initial cohort, for which genomic DNA was available even after the Infinium assay, were subjected to anion-exchange HPLC analysis. PCR products generated from the bisulfite-treated genomic DNA of each sample were measured along with the 0% and 100% control DNA fragments as described previously [23]. The representative chromatograms for the 5 marker CpG sites are shown in Fig. 2A, and those encompassing cg09580822 and cg15050398 showed chromatograms with an apparent single peak. The relative DNA methylation rate for each sample was then calculated proportionally by applying the retention time of the peak of each sample to the standard curve based on the retention times of the 0% and 100% control DNA fragments. For cg18210511 and cg09580859, obvious differences in the chromatograms were observed between NLT and NASH-W: NLT showed a “bimodal” peak pattern, whereas NASH-W showed a “single” peak pattern (Fig. 2A). The “bimodal” peak pattern chromatogram was fitted to two structural peaks using our in-house algorithm, and the relative DNA methylation rate for the chromatogram was calculated by summing the two peaks according to the area under each. For cg13719443, the chromatogram for NLT showed a “single peak with a shoulder” (arrow in Fig. 2A) pattern and its relative DNA methylation rate was again calculated after fitting to the structural peaks. The relative DNA methylation rates of NASH-W samples based on HPLC were significantly different from those of NLT samples for cg09580822, cg15050398, cg18210511, cg09580859 and cg13719443 (*P* = 3.00 × 10^−13^, *P* = 2.72 × 10^−12^, *P* = 1.47 × 10^−6^, *P* = 9.11 × 10^−6^ and *P* = 4.72 × 10^−10^, respectively) (Fig. 2B), indicating the usefulness of the five analyses for discrimination of NASH-W from NLT, even on the basis of HPLC.

Next, ROC curves were again generated for the five PCR products based on their relative DNA methylation rates by HPLC analysis, and the Youden index was calculated as a cutoff value for discrimination of NASH-W from NLT. Sufficient sensitivity (85.3–90.0%) and specificity (73.0–93.3%) for such discrimination were obtained in the initial cohort (Table 4).

**Table 4:** Discrimination of non-cancerous liver tissue showing non-alcoholic steatohepatitis (NASH) from patients with hepatocellular carcinoma (NASH-W) from normal liver tissue (NLT) based on the relative DNA methylation rates determined by anion-exchange high-performance liquid chromatography analysis in the initial and validation cohorts

PCR product ID^a^	Probe ID^a^	Initial cohort (*n* = 50)				Validation cohort (*n* = 33)	
		Area under the curve	Cutoff value^b^	Sensitivity^c^	Specificity^d^	Sensitivity^c^	Specificity^d^
1	cg09580822	0.965	0.667	0.853	0.933	0.909	0.364
2	cg15050398	0.980	1.000	0.900	0.933	0.909	0.227
3	cg18210511	0.865	0.641	0.900	0.759	1.000	0.864
4	cg09580859	0.812	0.849	0.900	0.770	1.000	0.818
5	cg13719443	0.850	0.408	0.900	0.730	0.811	0.727

^a^PCR product ID encompassing five marker CpG (Infinium HumanMethylation450 BeadChip probe) sites is shown in Additional file 1: Table S2

^b^Youden index based on the receiver operating characteristic curve was used as the cutoff value for the discrimination

^c^Sensitivity was defined as the ratio of the number of tissue samples predicted to be NASH-W and/or carcinogenic risk-positive based on the criteria relative to the exact number of NASH-W samples

^d^Specificity was defined as the ratio of the number of tissue samples not predicted to be NASH-W and/or predicted to be carcinogenic risk-negative based on the criteria relative to the exact number of NLT samples. Markers for which sufficient sensitivity and specificity were validated in the validation cohort are underlined

### Validation of estimation ability using the validation cohort

**Fig. 3:**
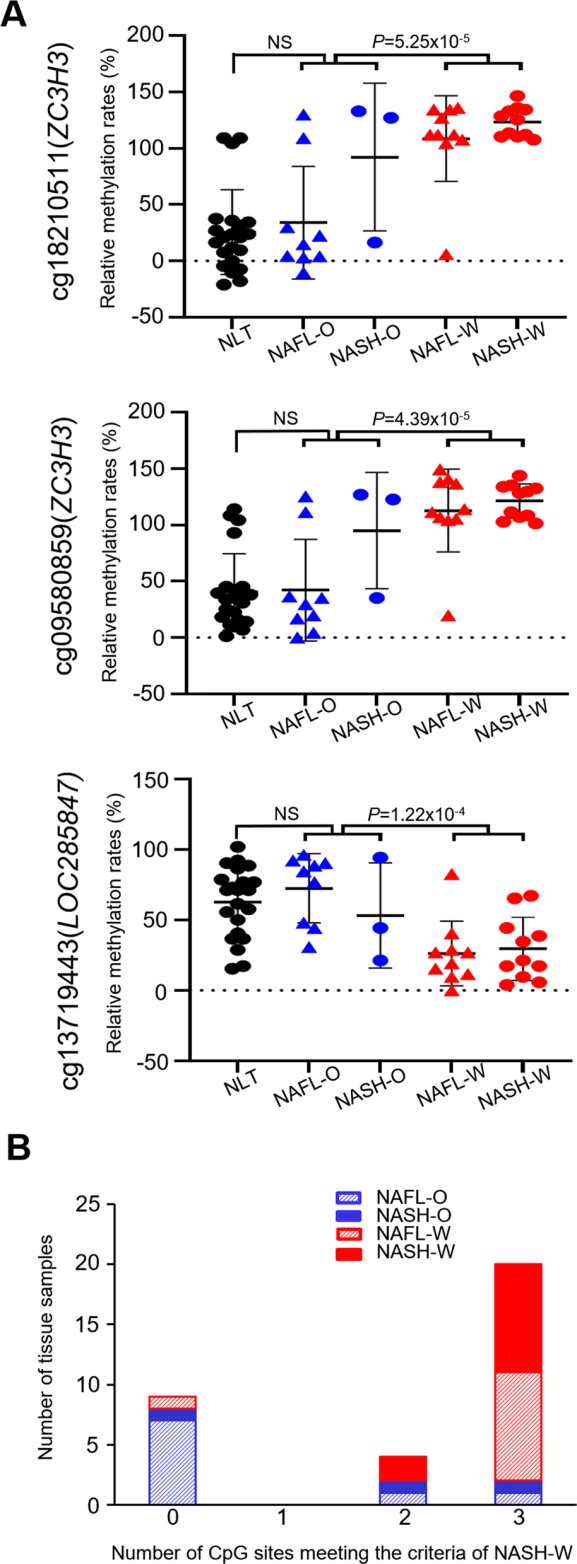
High-performance liquid chromatography (HPLC) analysis using the newly developed anion-exchange column for specimens of normal liver tissue (NLT) (*n* = 22, black), non-cancerous liver tissue (N) showing non-alcoholic fatty liver (NAFL) derived from partial hepatectomy specimens from patients without hepatocellular carcinoma (HCC) (NAFL-O) (*n* = 9, blue), *N* samples showing non-alcoholic steatohepatitis (NASH) from patients without HCC (NASH-O) (*n* = 3, blue), *N* samples showing NAFL from patients with HCC (NAFL-W) (*n* = 10, red), and *N* samples showing NASH from patients with HCC (NASH-W) (*n* = 11, red) in the validation cohort. **A** Scattergrams of the relative DNA methylation rates obtained by HPLC analysis for cg18210511, cg09580859 and cg13719443 whose estimation ability was validated in the validation cohort. Such rates were obtained as described in “Results” section. Such rates for both NAFL-W and NASH-W samples differed significantly from both NAFL-O and NASH-O samples regardless of histological features (i.e., regardless of NAFL or NASH), whereas the rates for both NAFL-O and NASH-O samples did not differ from those of NLT samples. *P* values were obtained by Welch’s *t* test. **B** Histograms showing the number of CpG sites satisfying the criteria for cg18210511, cg09580859 and cg13719443 based on the cutoff values shown in Table 4. If the tissue sample was estimated to have carcinogenic risk when two marker CpG sites or more satisfied the criteria for cg18210511, cg09580859 and cg13719443, regardless of histological features (i.e., regardless of NAFL or NASH), the liver tissue specimens from which HCCs had originated (9 NAFL-W samples and all 11 NASH-W samples) were considered to possess carcinogenic risk with 95% sensitivity. The possibility that patients from whom 2 NAFL-O and 2 NASH-O samples were obtained, which satisfied the criteria for two of more markers, would develop HCCs in the future could not be ruled out

In order to validate the estimation ability for NASH samples from which NASH-related HCCs had arisen, 22 NLT samples and 11 NASH-W samples in the validation cohort were subjected to anion-exchange HPLC analysis. Using the cutoff values set in the initial cohort and indicated in Table 4, sufficient sensitivity (81.1–100%) and specificity (72.7–86.4%) were confirmed in the validation cohort for the PCR products encompassing cg18210511, cg09580859 and cg13719443. As shown in Fig. 3A, the DNA methylation status of NASH-W samples (the origin of NASH-related HCC) clearly differed from that of NLT on cg18210511, cg09580859 and cg13719443 (*P* = 1.94 × 10^−9^, *P* = 1.78 × 10^−8^ and *P* = 7.78 × 10^−4^, respectively).

Moreover, the DNA methylation status of both NAFL-W samples and NASH-W samples differed significantly from that of NAFL-O samples and NASH-O samples on the basis of the three analyses, again indicating that tissue samples from which HCC has already arisen can be discriminated from tissue samples that have not yet generated HCC, regardless of their histological features (Fig. 3A). In addition, the DNA methylation status NAFL-O and NASH-O samples that had never generated HCC was similar to that of NLT samples, even though their histological features were compatible with NAFLD (Fig. 3A).

Finally, a histogram showing the numbers of tissue samples (NLT, NAFL-O, NASH-O, NAFL-W and NASH-W) subjected to HPLC analysis in the validation cohort and the numbers of marker CpG sites satisfying the criteria on cg18210511, cg09580859 and cg13719443 described in Table 4 was derived and is shown in Fig. 3B. If tissue samples satisfying the criteria for two or more markers after the three HPLC analyses were considered to have an inherent risk of carcinogenesis, then 95% of NAFL-W and NASH-W samples (9 NAFL-W samples and all 11 NASH-W samples) were deemed positive for carcinogenic risk in the validation cohort (Fig. 3B).

## Discussion

Our intention in the present study was to devise a protocol for carcinogenic risk estimation in liver biopsy specimens of patients with NASH based on quantification of DNA methylation, in order to facilitate early treatment of NASH-related HCCs through intensive surveillance and early detection. Identification of liver tissue specimens exhibiting histological features compatible with NASH and with the potential to give rise to HCCs, i.e., NASH-W samples, is considered to be the first step of carcinogenic risk estimation. In fact, the DNA methylation status of the 4050 CpG sites examined by Infinium assay clearly differed between NASH-W and NLT and allowed the two to be discriminated on the basis on this status (Fig. 1; Additional file 1: Table S3). The reliability of DNA methylation levels based on the Infinium assay was technically verified at representative CpG sites, i.e., the finally selected marker CpG sites cg18210511, cg09580859 and cg13719443, using pyrosequencing (see Additional file 1: Fig. S2). Since DNA methylation profiles in the precancerous NASH-W stage were inherited by or strengthened in the NASH-related HCCs themselves (Fig. 1), estimation of carcinogenic risk based on such profiles in NASH-W specimens appeared to be justified. Genes showing AUC values of more than 0.95 for discrimination of NASH-W samples from NLT samples were accumulated in molecular pathways participating in chromatin modification, autophagy, cell cycle regulation and DNA repair (Additional file 1: Table S4). It is quite feasible that disturbance of such pathways due to DNA methylation alterations would be of significance in the early risk stages of multistage NASH-related hepatocarcinogenesis.

In a clinical setting, diagnostic criteria that completely discriminate NASH-W from NASH-O may not be adequate, since some patients without HCCs at the time of NASH-O biopsy will ultimately develop HCCs if adequate intervention is not provided. Therefore, we focused on 21 candidate CpG sites (Table 2) and successfully identified NASH-W samples using our novel analytical method based on HPLC [23]. Although the NASH-O samples in the initial cohort had been obtained from morbidly obese patients, the DNA methylation levels of 20 of the 21 marker CpG sites were not significantly correlated with body mass index in any of the NASH-O and NASH-W patients in both the initial and validation cohorts (see Additional file 1: Table S8), indicating that, in general, differences in DNA methylation status between NASH-O and NASH-W are not attributable to obesity in NASH-O patients. Our criteria allowed identification of both NAFL-W and NASH-W as tissues from which HCC had originated (Fig. 3B), regardless of their histological features (Table 3), validating the novelty of our risk estimation criteria. Finally, the estimation ability of the HPLC-based system based on three markers was confirmed even in the validation cohort, suggesting the clinical feasibility of this approach for carcinogenic risk estimation in the near future.

The DNA methylation status of not only the exact Infinium probe CpG site but also neighboring multiple CpG sites—underlined in Additional file 1: Table S2—affects the results of HPLC-based analysis [23]. When the marker probe CpG sites identified by the Infinium assay were located within the stably controlled genomic domains wherein all CpG sites, i.e., the Infinium probe CpG site and its neighboring CpG sites, showed largely similar methylation alterations, the differences between NLTs and NASH-Ws were easy to clarify. Particularly in cg18210511 and cg09580859, the differences between NLTs and NASH-Ws were so clear that they were distinguishable by simply viewing the chromatograms, such as the “bimodal peak” pattern of NLT and the “single peak” pattern of NASH-W. Each structural peak fitting to bimodal peaks may reflect each of the cell lineages, such as hepatocytes and endothelial cells, constituting the liver tissue samples and showing different levels of methylation. In our analyses of cg18210511 and cg09580859, we assumed that alterations in the methylation levels of multiple CpG sites on the PCR products occurred in both cell lineages and resulted in a “single peak” chromatogram for the NASH-W samples. Moreover, we confirmed that the chromatogram patterns (single peak, single peak with a shoulder or bimodal peak) for most of the NASH-O samples were similar to those for the NLT samples, whereas the patterns for T samples were similar to those for the NASH-W samples (see Additional file 1: Fig. S3). Since our carcinogenic risk estimation can be performed with our HPLC-based system, which would be easy to introduce into hospital clinical laboratories, we would expect its clinical application to be feasible.

Marker Infinium probe CpG sites cg18210511 and cg09580859 were within a CpG island located in the body of the *ZC3H3* gene (Table 2). Recently, it has been clarified that zinc-finger protein ZC3H3 directly interacts with the MTR4-ZFC3H1 core dimer of the Poly(A) Tail eXosome Targeting (PAXT) connection [38]: Recruitment of the human ribonucleolytic RNA exosome to nuclear polyadenylated RNA is facilitated by the PAXT connection. Since loss of ZC3H3 reportedly results in accumulation of PAXT substrates, ZC3H3 is considered to play an important role in the turnover of nuclear pA + RNA [38]. Therefore, alterations of ZC3H3 expression may affect the post-transcriptional regulation of various tumor-related genes. Moreover, although the r value for the *ZC3H3* gene appears to be low in Additional file 1: Table S5 within the TCGA database, a positive correlation between DNA hypermethylation of CpG sites in the gene body of *ZC3H3* and its mRNA overexpression has been reported in tissue samples of human HCCs [39]. Our present findings and these previous reports suggest that DNA hypermethylation of the gene body CpG island of *ZC3H3* is present even from the precancerous NASH stage and participates in NASH-related multistage hepatocarcinogenesis via overexpression of ZC3H3, resulting in abnormal post-transcriptional regulation of various tumor-related genes.

The marker Infinium probe CpG site cg13719443 was located within a CpG island shelf in the gene body of the *LOC285847* gene (Table 2). The *LOC285847* gene has never been characterized in detail, and epigenetic regulation of its expression has not been reported. Therefore, even though reduced expression of *LOC285847* has been demonstrated using RNA-seq in blood samples from patients with proliferative diabetic retinopathy [40], the significance of cg13719443 hypomethylation during NASH-related hepatocarcinogenesis is difficult to interpret. Moreover, among the genes included in Table 2, only three showed a significant correlation between the levels of DNA methylation and expression (*P* < 0.05 and *r* < −0.2 or *r* > 0.2) (Additional file 1: Table S5). These findings are consistent with our previous studies, which revealed that even DNA methylation alterations at CpG sites not involved in regulating the expression of functionally important genes can become excellent surrogate markers for DNA methylation diagnostics [41, 42]. Particularly at the carcinogenic risk stage, but not in established cancers, it is feasible that DNA methylation alterations would not yet have expanded immediately to involve important tumor-related genes [19–22].

Biomarkers that are not affected by differences in age and sex would be useful in a clinical setting. Relative methylation rates determined by HPLC for cg18210511, cg09580859 and cg13719443 included in Fig. 3 did not differ significantly between male and female NASH patients (*n* = 34) in the initial and validation cohorts for whom HPLC data were obtained (*P* = 0.054, 0.066 and 0.715, respectively). Such rates for cg18210511and cg13719443 were not significantly correlated with age (*P* = 0.099 and 0.602, respectively), although the rates for cg09580859 showed marginal significance (*P* = 0.035). These data indicated that the DNA methylation status of our markers was, in general, not correlated with differences in age and sex, and that such markers would likely be useful irrespective of the age and sex of patients (see Additional file 1: Table S9).

In the validation study shown in Fig. 3, the small number of samples in the validation cohort would have been a limitation. On the other hand, we believe that a strong point of our study was that the reliability of our criteria was validated even in the validation cohort samples collected independently at a hospital other than the National Cancer Center where the initial cohort samples were collected. If a tissue sample is considered to have a risk of carcinogenesis when at least two of the markers cg18210511, cg09580859 and cg13719443 satisfy the criteria in Table 4, regardless of the severity of necroinflammation revealed by microscopy, then both NAFL-W and NASH-W can be considered positive for carcinogenic risk with 95% sensitivity (Fig. 3B). Although the DNA methylation rates of cg18210511 and cg09580859 cannot precisely discriminate NAFL-O and NASH-O samples from NASH-W samples at the present time (Fig. 3A) and 4 samples of NAFL-O and NASH-O were risk-positive, as shown in Fig. 3B, this may not have represented false positivity. NAFL-O and NASH-O patients whose samples overlapped the data obtained in the NASH-W groups may have been at risk of carcinogenesis, even though HCC had not developed at the time of sample collection. Therefore, we will need to perform long-term follow-up of these patients overlapping with NASH-W to identify any potential carcinogenesis risk that has not yet been demonstrated. Although prospective validation using a larger cohort of liver biopsy specimens will, of course, be needed, because the discrimination was based on our HPLC-based system which would be easy to introduce into hospital clinical laboratories [23], such carcinogenic risk estimation would be clinically applicable for personalized therapy of patients with NASH.

Another very interesting question is whether DNA methylation abnormalities related to carcinogenesis risk can be detected even in blood samples, and how long before the onset of HCC such abnormalities becomes detectable using such samples. If DNA methylation abnormalities in our marker CpG sites were to be detectable in blood samples long before the onset of HCC, repeated blood tests might offer a means of noninvasive carcinogenesis risk diagnosis to patients without a need for liver biopsy. Since the results of epigenome-wide association studies to detect disease-related DNA methylation abnormalities in blood samples have been reported recently [43], we assume that such noninvasive carcinogenesis risk diagnosis might be possible. Further DNA methylation analysis using blood samples obtained at various time points before the onset of HCC should be performed in the near future.

## Supplementary Information

**Additional file 1. Supplementary Table S1**. Clinicopathological characteristics of patients in the initial and validation cohorts.** Supplementary Table S2**. Primer sequences and PCR products for high-performance liquid chromatography.** Supplementary Figure S1**. Venn diagram showing the numbers of common and individual differentially methylated probes (P<0.05 after Bonferroni correction and |Δβ|≥0.1) based on the Infinium assay among samples of normal liver tissue (NLT), non-cancerous liver tissue showing non-alcoholic steatohepatitis (NASH) from partial hepatectomy specimens from patients without hepatocellular carcinoma (HCC) (NASH-O), non-cancerous liver tissue showing NASH from partial hepatectomy specimens from patients with HCC (NASH-W) and cancerous tissue (T).** Supplementary Table S3**. The 415 CpG sites for which receiver operating characteristic curve analysis showed area under the curve (AUC) values larger than 0.95 for discrimination of non-cancerous liver tissue showing non-alcoholic steatohepatitis (NASH) derived from partial hepatectomy specimens from patients with hepatocellular carcinoma (NASH-W) from normal liver tissue (NLT) samples. **Supplementary Table S4.** Seventy statistically significant pathway maps in which the 415 probes showing area under the curve (AUC) values more than 0.95 for discrimination of non-cancerous liver tissue samples showing non-alcoholic steatohepatitis from patients with hepatocellular carcinoma (NASH-W) from normal liver tissue (NLT) samples, and were designed for the 339 genes, were significantly (P<0.05) accumulated, as revealed by Reactome pathway analysis (https://reactome.org).** Supplementary Table S4**. Seventy statistically significant pathway maps in which the 415 probes showing area under the curve (AUC) values more than 0.95 for discrimination of non-cancerous liver tissue samples showing non-alcoholic steatohepatitis from patients with hepatocellular carcinoma (NASH-W) from normal liver tissue (NLT) samples, and were designed for the 339 genes, were significantly (P<0.05) accumulated, as revealed by Reactome pathway analysis (https://reactome.org).** Supplementary Table S5**. Correlation between levels of DNA methylation and expression for the 13 genes included in Table 2 and for which expression data are available in the The Cancer Genome Atlas database (https://www.cancer.gov/aboutnci/organization/ccg/research/structural-genomics/tcga).** Supplementary Table S6**. Comparison of DNA methylation levels based on the Infinium assay between samples of normal healthy liver tissue (GSE107038) deposited in the GEO database (https://www.ncbi.nlm.nih.gov/geo/query/acc.cgi?acc=GSE107038) and non-cancerous liver tissue samples showing non-alcoholic steatohepatitis from our cohort of patients with hepatocellular carcinoma (NASH-W).** Supplementary Table S7**. Multivariate analysis using the 91 samples of non-cancerous liver tissue showing non-alcoholic steatohepatitis (NASH) derived from biopsy specimens of patients with morbid obesity but without hepatocellular carcinoma (HCC) (NASH-O samples) and the 22 samples of non-cancerous liver tissue showing NASH derived from partial hepatectomy specimens of patients with HCC (NASH-W samples) in the initial cohorts.** Supplementary Figure S2**. Correlation between DNA methylation levels based on the Infinium assay and such levels based on pyrosequencing using samples of normal liver tissue (n=34), non-cancerous liver tissue showing non-alcoholic steatohepatitis from partial hepatectomy specimens from patients with hepatocellular carcinoma (n=22), and cancerous tissue samples (n = 22) in the initial cohort. Infinium data were successfully verified by pyrosequencing. (P<7.36×10-23 and Pearson correlation coefficient [r]>0.864).** Supplementary Table S8**. Correlation between DNA methylation levels based on the Infinium assay of the 21 marker CpG sites included in Table 2 and obesity in patients with nonalcoholic steatohepatitis (NASH) with and without hepatoceullar carcinoma in the initial and validation cohorts.** Supplementary Figure S3**. Representative chromatograms obtained by high-performance liquid chromatography analysis using a newly developed anion-exchange column for marker CpG sites in specimens of normal liver tissue (NLT), non-cancerous liver tissue (N) showing non-alcoholic steatohepatitis (NASH) derived from partial hepatectomy specimens frompatients without hepatocellular carcinoma (HCC) (NASH-O), N samples showing NASH from patients with HCC (NASH-W), and cancerous tissue (T) samples. The chromatogram patterns (bimodal peak or single peak with a shoulder [indicated by an arrow]) for most of the NASH-O samples are similar to those for NLT samples, whereas those for T samples (single peak) are similar to those for NASH-W samples.** Supplementary Table S9**. Correlation between relative methylation rates based on high-performance liquid chromatography (HPLC) and the sex and age of nonalcoholic steatohepatitis (NASH) patients with and without hepatoceullar carcinoma in the initial and validation cohorts from whom HLPC data had been obtained (n=34).

## Data Availability

Infinium data are deposited in the GEO database (https://www.ncbi.nlm.nih.gov/geo/, Accession numbers GSE89852 and GSE183468).

## Abbreviations

AUC: Area under the curve
DNMT1: DNA methyltransferase 1
NLT: Normal liver tissue
HBV: Hepatitis B virus
HCC: Hepatocellular carcinoma
HCV: Hepatitis C virus
HPLC: High-performance liquid chromatography
N: Non-cancerous liver tissue
NAFL: Non-alcoholic fatty liver
NAFLD: Non-alcoholic fatty liver disease
NAFL-O: Non-cancerous liver tissue showing non-alcoholic fatty liver from patients with liver metastasis of primary colorectal cancers but without hepatocellular carcinoma
NAFL-W: Non-cancerous liver tissue showing non-alcoholic fatty liver from patients with hepatocellular carcinoma
NAS: Non-alcoholic fatty liver disease activity score
NASH: Non-alcoholic steatohepatitis
NASH-O: Non-cancerous liver tissue showing non-alcoholic steatohepatitis from patients with liver metastasis of primary colorectal cancers but without hepatocellular carcinoma
NASH-W: Non-cancerous liver tissue showing non-alcoholic steatohepatitis from patients with hepatocellular carcinoma
PAXT: Poly(A) Tail eXosome Targeting
PCA: Principal component
ROC: Receiver operating characteristic
T: Cancerous tissue
TNM: Tumor–node–metastasis
