# Supplementary material for: Quantification of DNA methylation for carcinogenic risk estimation in patients with non-alcoholic steatohepatitis
Source: Clin Epigenetics. 2022 Dec 5;14:168. doi: 10.1186/s13148-022-01379-4 (PMC9724255; doi:10.1186/s13148-022-01379-4)
Supplement: Supplementary file 1 — Additional file 1. Supplementary Table S1. Clinicopathological characteristics of patients in the initial and validation cohorts. Supplementary Table S2. Primer sequences and PCR products for high-performance liquid chromatography. Supplementary Figure S1. Venn diagram showing the numbers of common and individual differentially methylated probes (P<0.05 after Bonferroni correction and |Δβ|≥0.1) based on the Infinium assay among samples of normal liver tissue (NLT), non-cancerous liver tissue showing non-alcoholic steatohepatitis (NASH) from partial hepatectomy specimens from patients without hepatocellular carcinoma (HCC) (NASH-O), non-cancerous liver tissue showing NASH from partial hepatectomy specimens from patients with HCC (NASH-W) and cancerous tissue (T). Supplementary Table S3. The 415 CpG sites for which receiver operating characteristic curve analysis showed area under the curve (AUC) values larger than 0.95 for discrimination of non-cancerous liver tissue showing non-alcoholic steatohepatitis (NASH) derived from partial hepatectomy specimens from patients with hepatocellular carcinoma (NASH-W) from normal liver tissue (NLT) samples. Supplementary Table S4. Seventy statistically significant pathway maps in which the 415 probes showing area under the curve (AUC) values more than 0.95 for discrimination of non-cancerous liver tissue samples showing non-alcoholic steatohepatitis from patients with hepatocellular carcinoma (NASH-W) from normal liver tissue (NLT) samples, and were designed for the 339 genes, were significantly (P<0.05) accumulated, as revealed by Reactome pathway analysis (https://reactome.org). Supplementary Table S4. Seventy statistically significant pathway maps in which the 415 probes showing area under the curve (AUC) values more than 0.95 for discrimination of non-cancerous liver tissue samples showing non-alcoholic steatohepatitis from patients with hepatocellular carcinoma (NASH-W) from normal liver tissue (NLT) samples, and were design [file 13148_2022_1379_MOESM1_ESM.pdf]

Additional file 1: Supplementary Table S1 Clinicopathological characteristics of patients in the initial and validation cohorts.

(A) Initial cohort

Parameters		Patient groups		
		Patients with colorectal cancer (CRC) (n=36)	Patients with morbid obesity (n=91)	Patients with hepatocellular carcinoma (HCC) (n=22)
Subjected tissue samples		Normal liver tissue (NLT) (n=36)	Non-cancerous liver tussue (N) showing non-alcoholic steatohepatitis (NASH) (NASH-O) (n=91)	N showing NASH (NASH-W) (n=22) and HCC (T) (n=22)
Clinicopathological features				
Age (years)		61.50 ± 8.05	42.49 ± 10.17	71.23 ± 7.65
Sex	Male	27	41	20
	Female	9	50	2
Histological findings in N				
Steatosis[a]	<5%	N/A	3	0
	5-33%	N/A	55	15
	33-66%	N/A	21	6
	>66%	N/A	11	1
Lobular inflammation[a]	None	N/A	1	0
	<2 foci per 200 x field	N/A	74	17
	2-4 foci per 200 x field	N/A	16	5
	>4 foci per 200 x field	N/A	0	0
Ballooning[a]	None	N/A	1	0
	Few	N/A	88	19
	Many	N/A	2	3
Fibrosis[b]	0	N/A	3	0
	1a	N/A	43	5
	1b	N/A	6	0
	1c	N/A	0	0
	2	N/A	22	10
	3	N/A	13	5
	4	N/A	4	2
NASH stage[b]	Grade 1	N/A	69	17
	Grade 2	N/A	18	4
	Grade 3	N/A	1	1
	Burned-out NASH	N/A	3	0
HCCs				
Tumor diameter (cm)		N/A	N/A	5.46 ± 2.66
Histological grade[c]	Well differentiated	N/A	N/A	3
	Moderately differentiated	N/A	NA	14
	Poorly differentiated	N/A	NA	5
TNM stage	I	N/A	NA	7
	II	N/A	NA	11
	IIIA	N/A	NA	2
	IIIB	N/A	NA	2
	IVA	N/A	NA	0
	IVB	N/A	N/A	0

(B) Validation cohort

Parameters		Patient groups				
		Patients with CRC (n=34)			Patients with HCC (n=21)	
Subjected tissue samples		NLT (n=22)	N showing non-alcoholic fatty liver (NAFL) (NAFL-O) (n=9)	NASH-O (n=3)	N showing NAFL (NAFL-W) (n=10)	NASH-W (n=11) and T (n=11)
Clinicopathological features						
Age (years)		58.77± 12.23	63.78± 11.72	60.33± 11.72	69.00±10.66	70.36±8.78
Sex	Male	13	7	2	8	8
	Female	9	2	1	2	3
Histological findings in N						
Steatosis[a]	<5%	N/A	0	0	0	0
	5-33%	N/A	9	2	10	6
	33-66%	N/A	0	1	0	4
	>66%	N/A	0	0	0	1
Lobular inflammation[a]	None	N/A	7	0	8	0
	<2 foci per 200 x field	N/A	2	0	0	4
	2-4 foci per 200 x field	N/A	0	3	2	7
	>4 foci per 200 x field	N/A	0	0	0	0
Ballooning[a]	None	N/A	9	0	10	0
	Few	N/A	0	3	0	11
	Many	N/A	0	0	0	0
Fibrosis[b]	0	N/A	6	0	1	0
	1a	N/A	2	1	0	3
	1b	N/A	0	1	0	1
	1c	N/A	1	0	5	0
	2	N/A	0	1	2	0
	3	N/A	0	0	2	7
	4	N/A	0	0	0	0
NASH stage[b]	Grade 1	N/A	N/A	1	NA	4
	Grade 2	N/A	N/A	2	NA	7
	Grade 3	N/A	N/A	0	NA	0
	Burned-out NASH	N/A	N/A	0	NA	0
HCCs						
Tumor diameter (cm)		N/A	N/A	N/A	7.14 ± 4.64	5.76 ± 2.95
Histological grade[c]	Well differentiated	N/A	N/A	N/A	2	3
	Moderately differentiated	N/A	N/A	N/A	7	7
	Poorly differentiated	N/A	N/A	N/A	1	1
TNM stage	I	N/A	N/A	N/A	0	0
	II	N/A	N/A	N/A	8	4
	IIIA	N/A	N/A	N/A	2	6
	IIIB	N/A	N/A	N/A	0	0
	IVA	N/A	N/A	N/A	0	1
	IVB	N/A	N/A	N/A	0	0

[a]Microscopic features (steatosis, lobular inflammation, ballooning and fibrosis) were evaluated according to the non-alcoholic fatty disease (NAFLD) activity score (Ref. 26). [b]NASH stage was defined according to the Brunt classification (Ref. 27). [c]Histological grade of HCCs was in accordance with the World Health Organization classification (Ref. 28). [d]TNM stage was defined according to the Union for International Cancer Control Tumor-Node-Metastasis classification (Ref. 29). N/A, not applicable.

Additional file 2: Supplementary Table S2 Primer sequences and PCR products for high-performance liquid chromatography.

PCR product ID	Probe ID[a]	Gene symbol	PCR primers (5'-3')		Target sequence (5'-3')[b]
			Forward	Reverse	
1	cg09580822	N/A	GGAAGTTGATTTGATGGGTAGTA	TCCAAAATAACTAATAAAAAAACATCC	GGAAGTTGATTTGATGGGCAGCACCGACCAAACCACGACTTATGAGGCTTGCAGATGCATGTCAGAATTAGGC<br>TGCTATGAAATACGGCGTGATCTGTTCCACCTTCTGTAAGTTTCTCAGAAGTGAGGACTCTACTCTCAGAAACA<br>GCGTCCTCCCTGCCACAGCCATCAATGTGCATGTGGTCGACACA**CG**CATTCACACACACTCACGGCTCATTAAA<br>ATGCGTTGTGAACTTGGCGTAATCCCCAGGATGTTTCTTCATTAGCTATTTTGGA
2	cg15050398	*LOC401242*	AAGTGTTTTTTTTGTAGGAGGG	TAAACTCCAAACTAAAACCTCTACC	AAGTGTTTCCTTCGTAGGAGGGC**CG**TAGGCAGCCATGGCGCCCAGCAGGAATGGCATGATGTTGAAGCCCCA<br>CTTCCACAAGGACTGGCAGCAGCGTGTGGCCACGTGGTTCAACCAGAAGATCCGCAGAATCAAGGCCCGGCA<br>AGCCAAAGGGCGTGCATCGCCCCGCGCCCGGAGAGTCGGGACCCATCTGGCCCATTGTGCTGTGCCCTGCT<br>GTGCGTTATCACATCAAGGTGCGCGCCGGCAGAGGCTTCAGCCTGGAGCTCA
3	cg18210511	*ZC3H3*	GGTAGGTAGGATTTGATTAGTGTTT	CTAAAATCTAATCAAACTATTATCCCCA	GGCAGGCAGGATCTGACCAGTGTTCACGTGTGCTCACACTGGCACC**CG**TGGGAAGGACAGGCGAGCGTGCC<br>GTGCTGTGTGCGTGCACATGCGCCACAGCGGCCCACACAGCAGCACGGGGCAAGCGGCCCGCCTCACCTTT<br>CACAGCCGCAGGCCCCACGTTCAGGCCACCTGGGGACAACAGCCTGACCAGACCCCAG
4	cg09580859	*ZC3H3*	GGTAAGATGAGTTTATGTGTTTTGA	TAAAATCTAATCAAACTATTATCCCCAA	GGTAAGATGAGCTCATGTGTCTTGAAAGCGCAAAGGTTAAAAATGAGTAAGCCTTGCATTCCTCGTTACCCCAG<br>GCCTGAGCACCCCACATCACAGCCGCAGAAACGGAGGCTCAGGCTGCCCGGAGGCAAGCCCCAAGCGGCAG<br>GCAGGATCTGACCAGTGTTCACGTGTGCTCACACTGGCACCCGTGGGAAGGACAGGCGAG**CG**TGCCGTGCT<br>GTGTGCGTGCACATGCGCCACAGCGGCCCACACAGCAGCACGGGGCAAGCGGCCCGCCTCACCTTTCACAG<br>CCGCAGGCCCCACGTTCAGGCCACCTGGGGACAACAGCCTGACCAGACCCCA
5	cg13719443	*LOC285847*	GTTTAGGGTAGGGTAATTTTTTTTTAG	CCAAACAAATACTATTAAAAATCCTCTAC	GTTTAGGGTAGGGTAATTTCCCCTTAGCTTCTATGCGCACAAGTGCTGGGCCGCTGCAGCGTCACCCTCGGG<br>AATGG**CG**GTGTCTTTTCGTAGAGGACCCCCAACAGTATCTGTCTGG

[a]Probe ID of the Infinium HumanMethylation450 BeadChip.

[b]Infinium probe CpG sites are shown in bold and all CpG sites in target sequences are underlined.

N/A, not annotated (designed in the intergenic regions).

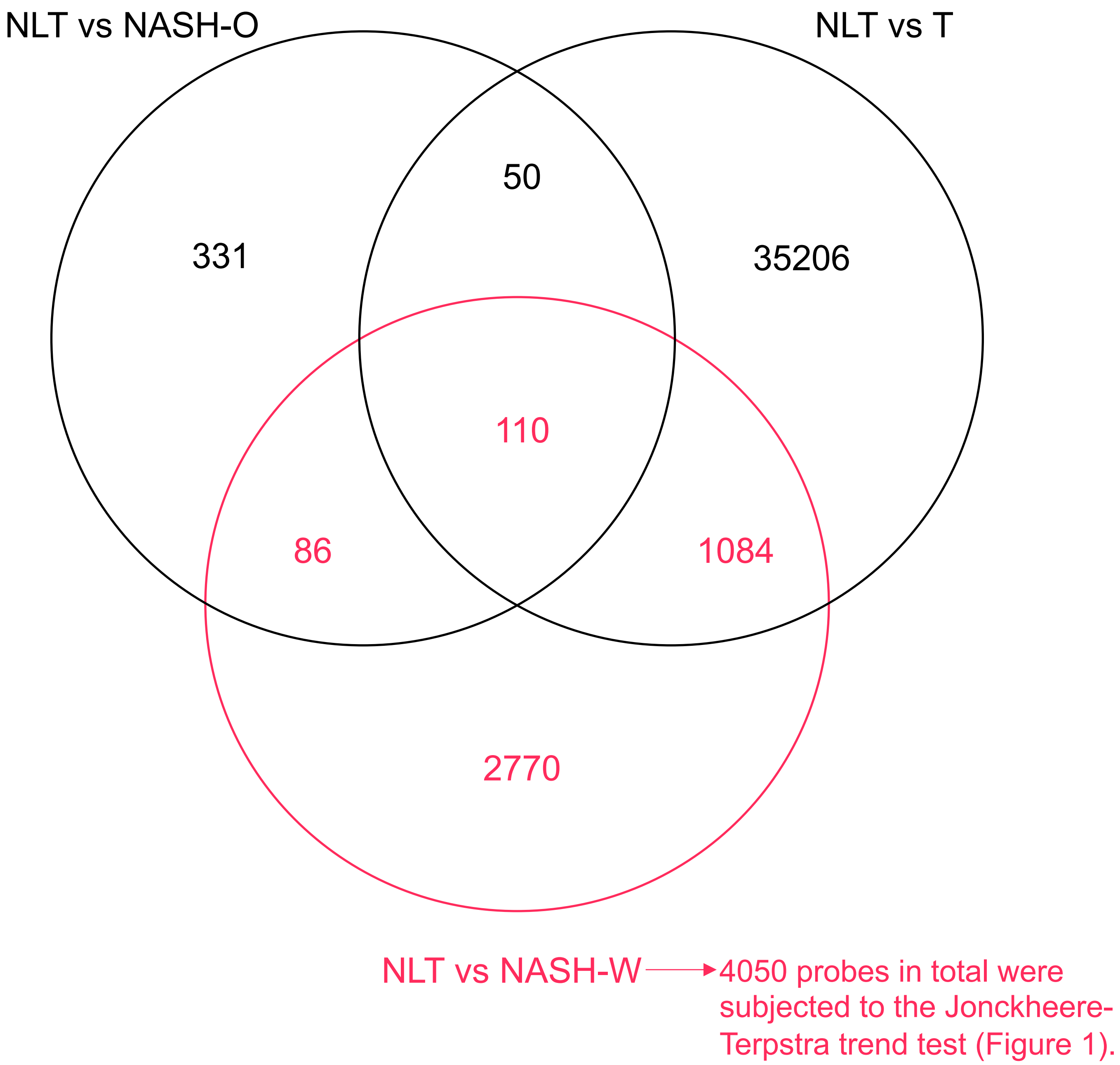

Additional file 3: Supplementary Figure S1 Venn diagram showing the numbers of common and individual differentially methylated probes ($P<0.05$ after Bonferroni correction and $|\Delta\beta|\geq 0.1$) based on the Infinium assay among samples of normal liver tissue (NLT), non-cancerous liver tissue showing non-alcoholic steatohepatitis (NASH) from partial hepatectomy specimens from patients without hepatocellular carcinoma (HCC) (NASH-O), non-cancerous liver tissue showing NASH from partial hepatectomy specimens from patients with HCC (NASH-W) and cancerous tissue (T).

Additional file 4: Supplementary Table S3 The 415 CpG sites for which receiver operating characteristic curve analysis showed area under the curve (AUC) values larger than 0.95 for discrimination of non-cancerous liver tissue showing non-alcoholic steatohepatitis (NASH) derived from partial hepatectomy specimens from patients with hepatocellular carcinoma (NASH-W) from normal liver tissue (NLT) samples.

Probe ID[a]	Gene symbol[b]	AUC	Cutoff value[c]	*P* value	Sensitivity[d]	Specificity[e]	DNA methylation status[f]
cg18457818	*RAN*	0.994	0.597	$3.27x10^{-7}$	0.972	0.955	NLT < NASH-W
cg19349861	*OPCML*	0.992	0.157	$2.57x10^{-12}$	1	0.909	NLT < NASH-W
cg07724654	*N/A*	0.99	0.841	$1.26x10^{-7}$	0.944	0.955	NLT < NASH-W
cg14427382	*PPP1R2P1*	0.99	0.654	$8.75x10^{-10}$	0.917	1	NLT > NASH-W
cg21980338	*ZNF141*	0.99	0.3	$1.44x10^{-9}$	0.944	1	NLT < NASH-W
cg24147392	*LOC440461*	0.99	0.306	$4.11x10^{-4}$	0.944	0.955	NLT < NASH-W
cg03505117	*N/A*	0.987	0.128	$3.61x10^{-2}$	0.944	0.955	NLT < NASH-W
cg15606745	*CACNG2*	0.987	0.609	$1.17x10^{-8}$	0.944	0.955	NLT > NASH-W
cg16319310	*N/A*	0.987	0.606	$8.55x10^{-11}$	1	0.909	NLT > NASH-W
cg03571507	*ZNF611*	0.986	0.719	$2.56x10^{-6}$	0.944	0.955	NLT > NASH-W
cg12424965	*EXOC2*	0.986	0.857	$4.43x10^{-4}$	0.972	0.955	NLT < NASH-W
cg14381908	*RASGEF1A*	0.986	0.296	$2.21x10^{-7}$	0.889	1	NLT < NASH-W
cg25824760	*ANKRD30B*	0.986	0.264	$5.18x10^{-14}$	0.889	1	NLT < NASH-W
cg20426671	*PITPNA*	0.985	0.496	$8.97x10^{-11}$	0.944	1	NLT < NASH-W
cg14299696	*N/A*	0.984	0.799	$3.43x10^{-9}$	0.914	1	NLT < NASH-W
cg12074375	*N/A*	0.984	0.806	$1.09x10^{-6}$	0.972	0.909	NLT > NASH-W
cg21152376	*N/A*	0.984	0.313	$1.00x10^{-2}$	0.917	1	NLT < NASH-W
cg25987208	*N/A*	0.983	0.271	$7.30x10^{-5}$	0.972	0.905	NLT < NASH-W
cg07076175	*FAM150A*	0.982	0.263	$1.94x10^{-2}$	0.944	1	NLT < NASH-W
cg07738077	*ZNF28*	0.982	0.601	$6.17x10^{-11}$	0.917	1	NLT > NASH-W
cg07886142	*MEGF10*	0.982	0.694	$7.85x10^{-8}$	0.917	0.955	NLT > NASH-W
cg11070069	*N/A*	0.982	0.183	$1.14x10^{-2}$	0.917	1	NLT < NASH-W
cg11213199	*MRPS24*	0.982	0.426	$6.98x10^{-13}$	0.861	1	NLT < NASH-W
cg12809925	*PARK7*	0.982	0.31	$3.06x10^{-5}$	1	0.909	NLT < NASH-W
cg22083325	*LOC440461*	0.982	0.245	$2.61x10^{-4}$	1	0.864	NLT < NASH-W
cg07138452	*FLCN*	0.981	0.203	$2.65x10^{-9}$	0.944	0.909	NLT < NASH-W
cg07956857	*N/A*	0.981	0.673	$1.03x10^{-10}$	0.972	0.909	NLT > NASH-W
cg07959490	*CCDC57*	0.981	0.532	$1.67x10^{-9}$	0.917	0.955	NLT < NASH-W
cg13161852	*N/A*	0.981	0.761	$2.39x10^{-10}$	0.972	0.909	NLT > NASH-W
cg13468249	*DULLARD*	0.981	0.265	$6.04x10^{-10}$	0.917	0.955	NLT < NASH-W
cg20251199	*SNORA14B*	0.981	0.264	$1.41x10^{-11}$	1	0.909	NLT < NASH-W
cg03666506	*TBC1D26*	0.98	0.654	$1.71x10^{-3}$	0.972	0.909	NLT > NASH-W
cg08081407	*ARF4*	0.98	0.213	$2.22x10^{-11}$	0.889	0.955	NLT < NASH-W
cg11036936	*RAD51L1*	0.98	0.646	$3.21x10^{-9}$	0.917	0.955	NLT > NASH-W
cg18855143	*LCE2B*	0.98	0.681	$4.09x10^{-2}$	0.972	0.909	NLT > NASH-W
cg21407906	*C20orf20*	0.98	0.256	$1.46x10^{-10}$	0.944	1	NLT < NASH-W
cg25512188	*RPS6KA2*	0.98	0.83	$2.29x10^{-5}$	0.972	0.909	NLT > NASH-W
cg01160882	*DKK1*	0.979	0.153	$6.65x10^{-6}$	0.861	1	NLT < NASH-W
cg16282892	*N/A*	0.979	0.625	$7.62x10^{-7}$	0.889	0.955	NLT > NASH-W
cg19003337	*KRT17*	0.979	0.714	$7.75x10^{-8}$	0.944	0.955	NLT > NASH-W
cg20592700	*WIPI2*	0.979	0.327	$7.16x10^{-10}$	0.889	0.955	NLT < NASH-W
cg23481024	*N/A*	0.979	0.61	1.39x10-19	0.889	0.955	NLT > NASH-W
cg14100946	*CDK6*	0.978	0.457	$2.03x10^{-5}$	0.971	0.909	NLT < NASH-W
cg10328259	*TSSC1*	0.977	0.738	$6.60x10^{-11}$	0.944	0.909	NLT > NASH-W
cg14648311	*N/A*	0.977	0.769	$4.43x10^{-10}$	0.889	0.955	NLT > NASH-W
cg20464143	*MYO10*	0.977	0.494	$4.09x10^{-8}$	1	0.864	NLT > NASH-W
cg02051077	*PODXL*	0.976	0.315	$3.35x10^{-3}$	1	0.864	NLT < NASH-W
cg05478392	*N/A*	0.976	0.426	$1.38x10^{-6}$	0.861	1	NLT < NASH-W
cg06361278	*N/A*	0.976	0.265	$3.40x10^{-9}$	1	0.818	NLT < NASH-W
cg10162914	*MFAP3L*	0.976	0.661	$4.75x10^{-8}$	0.944	0.909	NLT < NASH-W
cg15064753	*ZNF662*	0.976	0.664	$7.04x10^{-8}$	0.833	1	NLT > NASH-W
cg15284457	*N/A*	0.976	0.782	$1.79x10^{-5}$	0.972	0.955	NLT < NASH-W
cg15646741	*N/A*	0.976	0.457	$2.31x10^{-7}$	0.972	0.909	NLT > NASH-W

cg15920791	*N/A*	0.976	0.313	1.11×10^{-6}	0.972	0.909	NLT < NASH-W
cg18159180	*CUL7*	0.976	0.434	3.38×10^{-8}	0.861	1	NLT < NASH-W
cg20326359	*SNORD114-23*	0.976	0.823	3.38×10^{-4}	0.944	0.909	NLT > NASH-W
cg22216431	*NRIP2*	0.976	0.505	1.31×10^{-8}	0.944	0.955	NLT > NASH-W
cg22793065	*PCDHB4*	0.976	0.325	2.60×10^{-5}	0.944	0.909	NLT < NASH-W
cg02073839	*KIF25*	0.975	0.749	1.74×10^{-4}	0.972	0.909	NLT > NASH-W
cg03468541	*ZC3H14*	0.975	0.164	3.29×10^{-7}	0.972	0.864	NLT < NASH-W
cg04449562	*MYT1L*	0.975	0.73	2.35×10^{-2}	0.889	1	NLT > NASH-W
cg04867257	*N/A*	0.975	0.493	1.48×10^{-6}	0.972	0.909	NLT > NASH-W
cg05707844	*EIF2AK2*	0.975	0.25	4.80×10^{-9}	0.972	0.864	NLT < NASH-W
cg08941173	*PKNOX2*	0.975	0.356	2.25×10^{-10}	0.889	1	NLT < NASH-W
cg15936935	*PACS2*	0.975	0.611	5.70×10^{-7}	0.889	1	NLT > NASH-W
cg19105961	*CYB5D2*	0.975	0.332	9.24×10^{-10}	0.889	0.955	NLT < NASH-W
cg15470736	*UBE3C*	0.974	0.788	6.74×10^{-9}	1	0.905	NLT > NASH-W
cg00112042	*N/A*	0.973	0.192	1.22×10^{-7}	0.889	0.955	NLT < NASH-W
cg01593421	*N/A*	0.973	0.574	5.08×10^{-10}	0.833	1	NLT > NASH-W
cg03000596	*LAT2*	0.973	0.63	4.18×10^{-8}	0.917	0.955	NLT > NASH-W
cg03022552	*ZNF431*	0.973	0.781	2.60×10^{-7}	0.861	1	NLT > NASH-W
cg08912801	*N/A*	0.973	0.603	2.44×10^{-5}	0.972	0.909	NLT > NASH-W
cg09580822	*N/A*	0.973	0.682	2.01×10^{-3}	0.944	0.909	NLT > NASH-W
cg10526795	*H6PD*	0.973	0.68	4.39×10^{-10}	0.861	1	NLT > NASH-W
cg10824722	*N/A*	0.973	0.661	2.36×10^{-8}	0.889	0.955	NLT > NASH-W
cg12227411	*SNORD115-15*	0.973	0.682	9.36×10^{-7}	0.889	0.955	NLT > NASH-W
cg13178295	*N/A*	0.973	0.309	3.67×10^{-3}	0.889	0.955	NLT < NASH-W
cg13195185	*TNFRSF13B*	0.973	0.638	1.53×10^{-10}	0.917	0.955	NLT > NASH-W
cg18185028	*DHX36*	0.973	0.311	1.43×10^{-8}	0.889	0.955	NLT < NASH-W
cg24469923	*ZNF440*	0.973	0.094	1.07×10^{-6}	0.889	1	NLT < NASH-W
cg27248073	*FAM64A*	0.973	0.361	1.41×10^{-9}	0.917	0.909	NLT < NASH-W
cg00254681	*PRRT1*	0.972	0.205	1.39×10^{-7}	0.889	0.955	NLT < NASH-W
cg01073178	*TESC*	0.972	0.172	4.20×10^{-11}	0.889	1	NLT < NASH-W
cg01684248	*N/A*	0.972	0.414	1.59×10^{-10}	0.944	0.909	NLT < NASH-W
cg04294971	*RFNG*	0.972	0.299	1.57×10^{-3}	0.833	1	NLT < NASH-W
cg12313158	*GRM4*	0.972	0.574	4.83×10^{-8}	0.917	0.955	NLT > NASH-W
cg14497545	*MAML3*	0.972	0.604	9.04×10^{-11}	0.889	0.955	NLT > NASH-W
cg14977069	*LIME1*	0.972	0.439	1.07×10^{-9}	0.861	1	NLT < NASH-W
cg15473066	*ZNF727*	0.972	0.302	4.18×10^{-8}	0.889	1	NLT < NASH-W
cg16692538	*N/A*	0.972	0.568	3.85×10^{-2}	1	0.909	NLT > NASH-W
cg17810609	*N/A*	0.972	0.132	4.00×10^{-4}	0.944	0.909	NLT < NASH-W
cg19007908	*TRPM4*	0.972	0.628	2.13×10^{-7}	0.972	0.864	NLT < NASH-W
cg20673919	*PATE4*	0.972	0.469	3.64×10^{-10}	0.889	1	NLT > NASH-W
cg25220428	*N/A*	0.972	0.67	4.38×10^{-4}	0.833	1	NLT > NASH-W
cg03150409	*WHSC1*	0.971	0.717	2.62×10^{-11}	0.861	1	NLT > NASH-W
cg04176488	*MGC23284*	0.971	0.693	1.62×10^{-8}	0.833	1	NLT > NASH-W
cg04536393	*C16orf91*	0.971	0.486	2.25×10^{-7}	0.833	1	NLT > NASH-W
cg07576222	*SMAD3*	0.971	0.283	2.14×10^{-8}	0.889	1	NLT < NASH-W
cg08213792	*N/A*	0.971	0.648	3.59×10^{-9}	0.833	1	NLT > NASH-W
cg08524474	*ARHGAP22*	0.971	0.279	2.12×10^{-9}	0.917	0.955	NLT < NASH-W
cg13810766	*PRKAG2*	0.971	0.527	1.60×10^{-11}	0.861	0.955	NLT > NASH-W
cg14067770	*IRF7*	0.971	0.249	2.28×10^{-8}	0.833	1	NLT < NASH-W
cg14631053	*SSTR4*	0.971	0.288	5.06×10^{-3}	0.889	0.955	NLT < NASH-W
cg16357719	*RNPS1*	0.971	0.145	3.17×10^{-5}	0.917	0.955	NLT < NASH-W
cg18462141	*N/A*	0.971	0.672	1.59×10^{-9}	0.889	0.955	NLT > NASH-W
cg20615655	*FAM21B*	0.971	0.846	1.41×10^{-5}	0.861	1	NLT < NASH-W
cg24032568	*PPP2R2D*	0.971	0.874	8.33×10^{-4}	0.972	0.909	NLT < NASH-W
cg24153714	*C10orf11*	0.971	0.782	7.46×10^{-3}	0.861	0.955	NLT > NASH-W
cg27435660	*FBXO18*	0.971	0.714	8.83×10^{-10}	0.944	0.955	NLT > NASH-W
cg13551841	*EHMT1*	0.97	0.745	1.33×10^{-6}	1	0.909	NLT < NASH-W
cg00336022	*N/A*	0.97	0.735	1.42×10^{-5}	0.972	0.909	NLT < NASH-W

cg03973663	*LYN*	0.97	0.401	$4.36x10^{-8}$	0.889	1	NLT > NASH-W
cg04101934	*N/A*	0.97	0.488	$9.49x10^{-7}$	0.861	0.955	NLT < NASH-W
cg05211868	*N/A*	0.97	0.497	$1.43x10^{-8}$	0.833	1	NLT > NASH-W
cg06808467	*LOC339290*	0.97	0.181	$4.02x10^{-10}$	0.889	1	NLT < NASH-W
cg06959657	*N/A*	0.97	0.695	$6.20x10^{-9}$	0.972	0.909	NLT > NASH-W
cg08679807	*ACOT7*	0.97	0.709	$1.54x10^{-8}$	1	0.818	NLT > NASH-W
cg16678686	*N/A*	0.97	0.706	$2.57x10^{-6}$	0.917	0.909	NLT > NASH-W
cg22958118	*N/A*	0.97	0.653	$2.53x10^{-6}$	0.861	1	NLT > NASH-W
cg23770142	*APOM*	0.97	0.791	$1.78x10^{-7}$	0.917	0.909	NLT < NASH-W
cg24893198	*N/A*	0.97	0.865	$1.05x10^{-8}$	0.944	0.955	NLT < NASH-W
cg26272214	*FAM5B*	0.97	0.652	$4.01x10^{-3}$	0.889	1	NLT > NASH-W
cg27654966	*CUX1*	0.97	0.558	$4.29x10^{-8}$	0.944	0.909	NLT < NASH-W
cg01775802	*RGS6*	0.968	0.608	$1.53x10^{-9}$	0.917	0.909	NLT > NASH-W
cg01870519	*EXOC2*	0.968	0.634	$1.91x10^{-7}$	0.917	0.909	NLT > NASH-W
cg02173067	*N/A*	0.968	0.419	$1.14x10^{-3}$	0.833	1	NLT < NASH-W
cg02642298	*PRDXDD1P*	0.968	0.382	$1.75x10^{-7}$	0.889	1	NLT < NASH-W
cg03473408	*MRPS18B*	0.968	0.644	$1.06x10^{-5}$	0.972	0.909	NLT < NASH-W
cg04473235	*N/A*	0.968	0.617	$8.13x10^{-10}$	0.861	0.955	NLT > NASH-W
cg07737810	*N/A*	0.968	0.596	$1.08x10^{-7}$	0.944	0.864	NLT > NASH-W
cg09352908	*N/A*	0.968	0.835	$1.66x10^{-9}$	0.889	0.955	NLT > NASH-W
cg11964099	*N/A*	0.968	0.407	$6.59x10^{-7}$	0.861	1	NLT < NASH-W
cg23084309	*MLLT1*	0.968	0.238	$1.69x10^{-9}$	0.917	0.955	NLT < NASH-W
cg26330063	*FAM105A*	0.968	0.629	$3.28x10^{-8}$	0.806	1	NLT > NASH-W
cg26894854	*SLC45A3*	0.968	0.493	$5.46x10^{-4}$	0.972	0.864	NLT < NASH-W
cg00287477	*ACSL1*	0.967	0.27	$4.26x10^{-2}$	0.917	0.955	NLT < NASH-W
cg02524863	*N/A*	0.967	0.445	$1.82x10^{-5}$	0.889	0.955	NLT > NASH-W
cg06046629	*CACNA1D*	0.967	0.619	$4.39x10^{-10}$	0.833	1	NLT > NASH-W
cg06366345	*SNORD114-31*	0.967	0.755	$9.16x10^{-9}$	0.861	1	NLT > NASH-W
cg09741240	*NQO2*	0.967	0.167	$1.03x10^{-7}$	0.889	0.909	NLT < NASH-W
cg11311843	*PHOSPHO1*	0.967	0.308	$1.91x10^{-2}$	0.889	1	NLT < NASH-W
cg11677260	*N/A*	0.967	0.176	$2.29x10^{-4}$	0.833	1	NLT < NASH-W
cg12417549	*N/A*	0.967	0.634	$1.15x10^{-8}$	0.917	0.955	NLT > NASH-W
cg14094333	*KLF3*	0.967	0.264	$3.79x10^{-8}$	0.861	1	NLT < NASH-W
cg15465107	*N/A*	0.967	0.62	$7.48x10^{-11}$	0.917	0.955	NLT > NASH-W
cg19456996	*GABBR1*	0.967	0.208	$6.42x10^{-7}$	0.861	0.955	NLT < NASH-W
cg00593414	*N/A*	0.966	0.639	$1.02x10^{-6}$	1	0.818	NLT > NASH-W
cg01026613	*TMEM214*	0.966	0.521	$1.08x10^{-5}$	0.889	0.909	NLT < NASH-W
cg01400712	*EFR3B*	0.966	0.418	$1.17x10^{-7}$	0.889	1	NLT < NASH-W
cg06203912	*N/A*	0.966	0.745	$1.53x10^{-10}$	0.944	0.909	NLT > NASH-W
cg10420223	*UBD*	0.966	0.584	$2.78x10^{-9}$	0.972	0.955	NLT > NASH-W
cg12387604	*ANKRD17*	0.966	0.31	$1.91x10^{-9}$	0.861	0.955	NLT < NASH-W
cg14317230	*MTDH*	0.966	0.884	$5.23x10^{-5}$	0.972	0.818	NLT < NASH-W
cg16636571	*GYPB*	0.966	0.604	$1.85x10^{-6}$	0.861	1	NLT > NASH-W
cg17218154	*KIAA0907*	0.966	0.612	$1.36x10^{-7}$	0.944	0.864	NLT < NASH-W
cg17476752	*N/A*	0.966	0.563	$1.01x10^{-8}$	0.806	1	NLT > NASH-W
cg17499729	*N/A*	0.966	0.588	$4.74x10^{-8}$	0.861	0.955	NLT > NASH-W
cg17847344	*EBF1*	0.966	0.387	$9.05x10^{-5}$	0.972	0.864	NLT < NASH-W
cg20390253	*C18orf22*	0.966	0.308	$4.05x10^{-7}$	0.917	1	NLT < NASH-W
cg25738236	*BRCA1*	0.966	0.698	$1.26x10^{-7}$	0.861	0.955	NLT > NASH-W
cg00987699	*WIPI2*	0.965	0.199	$2.14x10^{-11}$	0.944	0.955	NLT < NASH-W
cg02531277	*CUX1*	0.965	0.613	$2.96x10^{-6}$	0.917	0.909	NLT < NASH-W
cg04396850	*N/A*	0.965	0.691	$5.30x10^{-6}$	0.944	0.909	NLT > NASH-W
cg05231098	*SMARCA4*	0.965	0.489	$4.90x10^{-8}$	0.861	1	NLT < NASH-W
cg08707819	*RCOR1*	0.965	0.363	$1.30x10^{-8}$	0.917	0.909	NLT < NASH-W
cg11397033	*N/A*	0.965	0.769	$5.57x10^{-5}$	0.917	0.909	NLT > NASH-W
cg12659254	*FBRSL1*	0.965	0.606	$2.55x10^{-9}$	0.944	0.864	NLT > NASH-W
cg13260314	*N/A*	0.965	0.651	$1.09x10^{-7}$	0.806	1	NLT > NASH-W
cg13896783	*CCDC148*	0.965	0.337	$1.53x10^{-9}$	0.861	1	NLT < NASH-W

cg14880282	*LOC284232*	0.965	0.624	$5.69x10^{-5}$	0.889	0.909	NLT > NASH-W
cg19933985	*N/A*	0.965	0.788	$4.85x10^{-8}$	0.889	0.909	NLT > NASH-W
cg20769334	*SBF1*	0.965	0.202	$2.78x10^{-8}$	0.889	0.955	NLT < NASH-W
cg20809690	*TNIP2*	0.965	0.369	$6.58x10^{-9}$	0.861	0.955	NLT < NASH-W
cg23129217	*N/A*	0.965	0.735	$1.51x10^{-5}$	0.889	0.955	NLT > NASH-W
cg23601374	*HDAC4*	0.965	0.55	$4.16x10^{-5}$	0.944	0.909	NLT < NASH-W
cg26112390	*GOLGA2L1*	0.965	0.721	$1.17x10^{-6}$	1	0.818	NLT > NASH-W
cg05310990	*ZNF85*	0.964	0.16	$2.23x10^{-6}$	0.806	1	NLT < NASH-W
cg04584009	*REXO1*	0.963	0.441	$5.13x10^{-7}$	0.917	0.909	NLT < NASH-W
cg04662828	*N/A*	0.963	0.525	$1.86x10^{-6}$	1	0.818	NLT > NASH-W
cg05703502	*KIAA2018*	0.963	0.506	$8.60x10^{-3}$	0.806	1	NLT < NASH-W
cg05909891	*N/A*	0.963	0.642	$9.87x10^{-9}$	0.861	0.955	NLT > NASH-W
cg08579753	*N/A*	0.963	0.534	$6.88x10^{-9}$	0.861	0.955	NLT > NASH-W
cg09570855	*N/A*	0.963	0.535	$1.11x10^{-6}$	0.778	1	NLT > NASH-W
cg10875261	*N/A*	0.963	0.811	$3.36x10^{-7}$	0.917	0.864	NLT > NASH-W
cg15617548	*GORASP2*	0.963	0.169	$2.67x10^{-8}$	0.806	1	NLT < NASH-W
cg15888301	*NEO1*	0.963	0.145	$1.57x10^{-5}$	0.917	0.955	NLT < NASH-W
cg16157055	*N/A*	0.963	0.578	$1.64x10^{-8}$	0.861	0.955	NLT > NASH-W
cg19861632	*GPR125*	0.963	0.545	$3.86x10^{-3}$	0.944	0.909	NLT < NASH-W
cg20943076	*SDK1*	0.963	0.757	$4.85x10^{-6}$	0.889	0.955	NLT > NASH-W
cg21530210	*N/A*	0.963	0.638	$1.11x10^{-6}$	0.972	0.909	NLT > NASH-W
cg23108607	*KIAA0754*	0.963	0.751	$4.54x10^{-7}$	0.861	0.955	NLT > NASH-W
cg25444615	*N/A*	0.963	0.374	$3.74x10^{-9}$	0.889	0.909	NLT > NASH-W
cg10187692	*VPS13B*	0.963	0.246	$4.18x10^{-3}$	0.861	1	NLT < NASH-W
cg00470768	*INO80*	0.962	0.583	$3.48x10^{-5}$	0.972	0.818	NLT > NASH-W
cg00731201	*N/A*	0.962	0.583	$4.03x10^{-4}$	0.917	0.955	NLT > NASH-W
cg01601050	*STXBP6*	0.962	0.233	$1.35x10^{-8}$	0.917	0.909	NLT > NASH-W
cg03196364	*BEST3*	0.962	0.538	$1.77x10^{-5}$	0.972	0.864	NLT > NASH-W
cg03321231	*RPH3AL*	0.962	0.711	$1.31x10^{-7}$	0.889	0.909	NLT > NASH-W
cg05526438	*N/A*	0.962	0.703	$8.17x10^{-9}$	0.917	0.955	NLT > NASH-W
cg07661849	*ZNF827*	0.962	0.643	$4.77x10^{-4}$	0.806	1	NLT > NASH-W
cg07867325	*N/A*	0.962	0.671	$2.42x10^{-8}$	0.889	0.955	NLT > NASH-W
cg08365438	*N/A*	0.962	0.654	$1.04x10^{-8}$	0.778	1	NLT > NASH-W
cg09622330	*GRIN2A*	0.962	0.77	$3.15x10^{-7}$	0.861	0.955	NLT > NASH-W
cg11855759	*USP43*	0.962	0.191	$1.00x10^{-8}$	0.889	0.955	NLT < NASH-W
cg12887985	*N/A*	0.962	0.187	$3.01x10^{-7}$	0.917	0.909	NLT < NASH-W
cg13435243	*LRRC8A*	0.962	0.858	$1.04x10^{-3}$	0.889	0.909	NLT < NASH-W
cg23322242	*N/A*	0.962	0.491	$4.23x10^{-11}$	0.806	1	NLT > NASH-W
cg23785719	*STK17A*	0.962	0.268	$8.84x10^{-8}$	0.944	0.909	NLT < NASH-W
cg24016617	*N/A*	0.962	0.625	$1.00x10^{-5}$	0.833	0.952	NLT > NASH-W
cg10475689	*RUNDC2A*	0.961	0.6	$1.45x10^{-8}$	0.829	1	NLT > NASH-W
cg01228685	*PLEC1*	0.961	0.659	$3.90x10^{-5}$	0.972	0.818	NLT > NASH-W
cg03916189	*N/A*	0.961	0.417	$1.99x10^{-4}$	0.972	0.909	NLT > NASH-W
cg04800503	*HOXB3*	0.961	0.409	$1.90x10^{-6}$	0.861	0.955	NLT < NASH-W
cg07797518	*LRRFIP1*	0.961	0.334	$5.53x10^{-6}$	0.944	0.864	NLT < NASH-W
cg09789536	*KLHL17*	0.961	0.308	$3.90x10^{-8}$	0.889	0.955	NLT < NASH-W
cg13041389	*N/A*	0.961	0.532	$2.40x10^{-5}$	0.972	0.909	NLT > NASH-W
cg13231117	*N/A*	0.961	0.757	$1.28x10^{-7}$	0.861	0.955	NLT > NASH-W
cg15615645	*C10orf26*	0.961	0.413	$1.28x10^{-3}$	0.917	0.909	NLT < NASH-W
cg15828524	*CACNB3*	0.961	0.738	$1.61x10^{-7}$	0.889	0.909	NLT < NASH-W
cg19569340	*RNF216*	0.961	0.427	$1.15x10^{-7}$	0.917	0.955	NLT < NASH-W
cg23113715	*N/A*	0.961	0.288	$1.52x10^{-9}$	0.861	1	NLT < NASH-W
cg23713156	*WDR45L*	0.961	0.193	$6.56x10^{-8}$	0.806	1	NLT < NASH-W
cg25548582	*UTP14C*	0.961	0.767	$2.19x10^{-4}$	0.917	1	NLT > NASH-W
cg00431813	*C7orf50*	0.96	0.542	$1.38x10^{-6}$	0.944	0.909	NLT < NASH-W
cg01779076	*CFLAR*	0.96	0.332	$4.44x10^{-7}$	0.889	0.909	NLT < NASH-W
cg02510736	*ZCCHC24*	0.96	0.186	$1.38x10^{-9}$	0.833	1	NLT < NASH-W
cg02792602	*TNS1*	0.96	0.214	$7.17x10^{-8}$	0.833	1	NLT < NASH-W

cg04607442	*N/A*	0.96	0.179	3.33×10^{-8}	0.889	0.955	NLT < NASH-W
cg04680436	*N/A*	0.96	0.548	4.32×10^{-4}	0.944	0.909	NLT < NASH-W
cg04762129	*N/A*	0.96	0.552	1.58×10^{-7}	0.806	1	NLT > NASH-W
cg04786142	*N/A*	0.96	0.698	7.34×10^{-4}	0.917	0.909	NLT > NASH-W
cg05393736	*NRBF2*	0.96	0.701	1.43×10^{-6}	0.972	0.818	NLT < NASH-W
cg06356708	*RCC2*	0.96	0.822	6.33×10^{-6}	0.806	1	NLT < NASH-W
cg06606511	*N/A*	0.96	0.617	7.12×10^{-10}	0.806	1	NLT > NASH-W
cg08648997	*LRP5*	0.96	0.683	3.15×10^{-6}	0.972	0.909	NLT < NASH-W
cg10199913	*HIST1H3C*	0.96	0.164	9.70×10^{-5}	0.778	1	NLT < NASH-W
cg14520079	*N/A*	0.96	0.524	4.10×10^{-6}	0.917	0.864	NLT > NASH-W
cg14564724	*ABCA1*	0.96	0.446	7.75×10^{-5}	0.889	0.955	NLT < NASH-W
cg15574461	*SRC*	0.96	0.822	3.56×10^{-3}	0.944	0.864	NLT < NASH-W
cg16136385	*CKAP5*	0.96	0.284	8.63×10^{-9}	0.889	1	NLT < NASH-W
cg16920238	*CCDC57*	0.96	0.547	2.31×10^{-3}	0.889	0.955	NLT < NASH-W
cg17030628	*IL20*	0.96	0.461	4.00×10^{-4}	0.889	0.955	NLT < NASH-W
cg17030981	*ZNF876P*	0.96	0.715	5.54×10^{-9}	0.833	0.955	NLT > NASH-W
cg18371636	*POM121C*	0.96	0.716	7.48×10^{-8}	0.861	0.955	NLT > NASH-W
cg19513374	*ZNF311*	0.96	0.714	1.59×10^{-5}	0.778	1	NLT > NASH-W
cg21610999	*SPDYE4*	0.96	0.609	6.56×10^{-10}	0.889	1	NLT > NASH-W
cg21685006	*PPP1R2P1*	0.96	0.578	3.87×10^{-4}	0.917	0.909	NLT < NASH-W
cg22900519	*N/A*	0.96	0.778	1.46×10^{-8}	0.889	1	NLT > NASH-W
cg27056425	*HIST1H2AI*	0.96	0.147	3.50×10^{-4}	0.889	0.955	NLT < NASH-W
cg01864791	*BHLHE40*	0.958	0.571	2.15×10^{-2}	0.917	0.955	NLT < NASH-W
cg05596328	*N/A*	0.958	0.241	3.87×10^{-4}	0.944	0.909	NLT < NASH-W
cg05997362	*LYN*	0.958	0.428	5.05×10^{-7}	0.833	1	NLT > NASH-W
cg06375525	*ESPNL*	0.958	0.796	8.04×10^{-4}	0.861	1	NLT < NASH-W
cg10827434	*RPF1*	0.958	0.108	2.41×10^{-6}	0.833	1	NLT < NASH-W
cg11223184	*ANKRD44*	0.958	0.837	4.70×10^{-7}	0.778	1	NLT < NASH-W
cg14325661	*TRPM4*	0.958	0.646	7.36×10^{-7}	0.972	0.909	NLT < NASH-W
cg15050398	*N/A*	0.958	0.473	1.68×10^{-7}	1	0.773	NLT > NASH-W
cg15610437	*AZU1*	0.958	0.7	6.70×10^{-5}	0.944	0.909	NLT > NASH-W
cg16015499	*TAGLN2*	0.958	0.821	2.79×10^{-7}	0.889	0.955	NLT > NASH-W
cg16674116	*TOLLIP*	0.958	0.531	1.98×10^{-5}	0.917	0.909	NLT < NASH-W
cg17095167	*N/A*	0.958	0.795	6.14×10^{-8}	0.806	1	NLT > NASH-W
cg21285133	*ANP32E*	0.958	0.364	3.99×10^{-7}	0.972	0.818	NLT > NASH-W
cg21397124	*N/A*	0.958	0.562	2.54×10^{-5}	0.889	0.909	NLT > NASH-W
cg26528255	*TMEM222*	0.958	0.713	7.40×10^{-8}	0.861	0.955	NLT > NASH-W
cg14950303	*FOXD4L1*	0.957	0.519	2.88×10^{-6}	0.914	0.909	NLT < NASH-W
cg00109302	*N/A*	0.957	0.167	5.88×10^{-4}	0.917	0.955	NLT < NASH-W
cg01236961	*N/A*	0.957	0.615	1.08×10^{-6}	0.861	0.955	NLT > NASH-W
cg01346915	*UBE2I*	0.957	0.877	3.44×10^{-5}	0.889	0.909	NLT < NASH-W
cg02095553	*N/A*	0.957	0.62	1.50×10^{-6}	0.889	0.909	NLT > NASH-W
cg02536431	*TUBB*	0.957	0.606	7.59×10^{-6}	0.75	1	NLT > NASH-W
cg03734864	*PRR5L*	0.957	0.505	3.67×10^{-5}	0.889	0.909	NLT < NASH-W
cg04680494	*KIF25*	0.957	0.733	2.12×10^{-5}	0.861	0.955	NLT > NASH-W
cg05357347	*N/A*	0.957	0.785	4.90×10^{-5}	0.778	1	NLT > NASH-W
cg10530613	*TRIT1*	0.957	0.83	1.16×10^{-4}	0.917	0.955	NLT < NASH-W
cg10558740	*FLJ22536*	0.957	0.688	2.51×10^{-3}	0.944	0.864	NLT > NASH-W
cg10632328	*FLJ35220*	0.957	0.183	2.97×10^{-4}	0.972	0.818	NLT < NASH-W
cg11152384	*N/A*	0.957	0.429	1.38×10^{-6}	0.972	0.909	NLT > NASH-W
cg11218561	*N/A*	0.957	0.756	6.05×10^{-7}	0.833	1	NLT > NASH-W
cg13048147	*ATP5H*	0.957	0.594	1.76×10^{-5}	0.861	0.955	NLT < NASH-W
cg17341174	*BAIAP2L1*	0.957	0.504	1.92×10^{-5}	0.944	0.909	NLT < NASH-W
cg18545992	*NCRNA00164*	0.957	0.498	9.86×10^{-7}	1	0.818	NLT > NASH-W
cg20407713	*DLG2*	0.957	0.791	2.18×10^{-8}	0.917	0.909	NLT > NASH-W
cg21290290	*C1orf93*	0.957	0.165	3.46×10^{-6}	0.889	0.909	NLT < NASH-W
cg25187967	*N/A*	0.957	0.804	1.73×10^{-8}	0.833	1	NLT > NASH-W
cg26015681	*N/A*	0.957	0.481	1.64×10^{-3}	0.944	0.864	NLT > NASH-W

cg25367568	*GNAS*	0.956	0.671	$1.62x10^{-2}$	0.914	0.955	NLT > NASH-W
cg01079950	*N/A*	0.956	0.651	$3.13x10^{-2}$	0.944	0.864	NLT > NASH-W
cg01730258	*SNX32*	0.956	0.528	$9.00x10^{-5}$	0.917	0.909	NLT < NASH-W
cg01993208	*C9orf3*	0.956	0.42	$5.41x10^{-5}$	0.917	0.955	NLT < NASH-W
cg01994308	*PLAG1*	0.956	0.253	$2.02x10^{-8}$	0.861	0.955	NLT < NASH-W
cg02788013	*INPP5D*	0.956	0.662	$2.65x10^{-5}$	0.806	1	NLT > NASH-W
cg03008165	*N/A*	0.956	0.796	$9.94x10^{-9}$	0.861	0.955	NLT > NASH-W
cg03274739	*N/A*	0.956	0.844	$1.60x10^{-2}$	0.833	1	NLT > NASH-W
cg05060577	*N/A*	0.956	0.565	$3.20x10^{-3}$	0.833	1	NLT > NASH-W
cg05484985	*N/A*	0.956	0.637	$8.65x10^{-5}$	0.972	0.864	NLT < NASH-W
cg05742286	*N/A*	0.956	0.704	$1.35x10^{-8}$	0.889	0.909	NLT > NASH-W
cg07628073	*N/A*	0.956	0.611	$1.28x10^{-7}$	0.944	0.909	NLT < NASH-W
cg07762785	*ZNF486*	0.956	0.683	$1.30x10^{-6}$	0.944	0.909	NLT > NASH-W
cg08775137	*N/A*	0.956	0.736	$4.85x10^{-4}$	0.972	0.909	NLT > NASH-W
cg09686800	*IPMK*	0.956	0.679	$6,74x10^{-6}$	0.972	0.818	NLT < NASH-W
cg10752406	*AZU1*	0.956	0.592	$1.08x10^{-6}$	0.833	1	NLT > NASH-W
cg12828313	*N/A*	0.956	0.197	$1.37x10^{-9}$	0.889	0.955	NLT < NASH-W
cg18210511	*ZC3H3*	0.956	0.521	$1.20x10^{-5}$	0.889	0.909	NLT < NASH-W
cg19015611	*VAX2*	0.956	0.463	$3.57x10^{-10}$	0.806	1	NLT < NASH-W
cg19556901	*SNORD115-1*	0.956	0.789	$6.59x10^{-6}$	0.889	0.909	NLT > NASH-W
cg19955284	*SLC44A4*	0.956	0.609	$1.60x10^{-6}$	0.889	0.955	NLT < NASH-W
cg23157419	*PTPN9*	0.956	0.672	$1.39x10^{-6}$	0.833	1	NLT < NASH-W
cg23465426	*PPP1R2P1*	0.956	0.597	$3.17x10^{-7}$	0.806	1	NLT > NASH-W
cg23476209	*LOC100133315*	0.956	0.574	$1.72x10^{-4}$	0.861	1	NLT < NASH-W
cg24599650	*RPL8*	0.956	0.638	$1.48x10^{-5}$	0.889	0.909	NLT > NASH-W
cg27055601	*LAMP1*	0.956	0.504	$4.30x10^{-3}$	0.806	1	NLT < NASH-W
cg00236302	*RAP1B*	0.955	0.461	$4.57x10^{-7}$	0.889	0.955	NLT < NASH-W
cg03092271	*RPSAP58*	0.955	0.594	$1.67x10^{-6}$	0.914	0.864	NLT > NASH-W
cg04010872	*N/A*	0.955	0.752	$2.70x10^{-3}$	0.861	0.955	NLT > NASH-W
cg04892437	*TSNAX-DISC1*	0.955	0.494	$3.84x10^{-5}$	0.972	0.909	NLT < NASH-W
cg07102320	*N/A*	0.955	0.504	$1.02x10^{-6}$	0.944	0.864	NLT < NASH-W
cg07475178	*LDHD*	0.955	0.742	$3.75x10^{-7}$	0.944	0.909	NLT < NASH-W
cg09141413	*GPRC5B*	0.955	0.233	$8.96x10^{-6}$	0.972	0.818	NLT < NASH-W
cg10690677	*CLCA4*	0.955	0.843	$4.62x10^{-7}$	0.917	0.955	NLT > NASH-W
cg12538597	*N/A*	0.955	0.677	$2.11x10^{-7}$	0.861	0.955	NLT > NASH-W
cg14058010	*N/A*	0.955	0.31	$1.28x10^{-6}$	0.917	0.864	NLT > NASH-W
cg17424859	*N/A*	0.955	0.704	$9.04x10^{-7}$	0.861	0.955	NLT > NASH-W
cg19751754	*N/A*	0.955	0.564	$4.96x10^{-6}$	0.889	0.864	NLT > NASH-W
cg21518151	*N/A*	0.955	0.628	$3.86x10^{-8}$	0.944	0.909	NLT > NASH-W
cg22685816	*MUC6*	0.955	0.505	$1.45x10^{-3}$	0.917	0.864	NLT < NASH-W
cg22921692	*FBXL12*	0.955	0.68	$2.19x10^{-7}$	0.889	0.909	NLT > NASH-W
cg24192846	*PPP1R11*	0.955	0.674	$5.74x10^{-9}$	0.861	0.955	NLT > NASH-W
cg24946729	*DTNA*	0.955	0.607	$2.24x10^{-5}$	0.889	0.955	NLT < NASH-W
cg26111913	*FARP1*	0.955	0.71	$2.46x10^{-7}$	0.889	0.909	NLT > NASH-W
cg27048142	*IGFL4*	0.955	0.674	$8.63x10^{-5}$	0.889	0.955	NLT > NASH-W
cg22306579	*ZBTB12*	0.955	0.385	$2.35x10^{-5}$	0.917	0.909	NLT < NASH-W
cg01214458	*MN1*	0.953	0.302	$1.84x10^{-3}$	0.833	0.955	NLT < NASH-W
cg01488306	*F13B*	0.953	0.61	$1.18x10^{-4}$	1	0.773	NLT > NASH-W
cg02072589	*N/A*	0.953	0.583	$9.33x10^{-7}$	0.861	0.955	NLT > NASH-W
cg02438164	*N/A*	0.953	0.52	$2.65x10^{-6}$	0.889	0.864	NLT > NASH-W
cg02596190	*BNIP2*	0.953	0.439	$2.19x10^{-5}$	1	0.818	NLT < NASH-W
cg03488113	*N/A*	0.953	0.685	$1.61x10^{-6}$	0.861	0.955	NLT > NASH-W
cg04628742	*HLA-J*	0.953	0.762	$1.34x10^{-3}$	0.833	1	NLT > NASH-W
cg05998816	*PCNT*	0.953	0.803	$6.79x10^{-6}$	0.972	0.818	NLT > NASH-W
cg06752157	*MYST4*	0.953	0.55	$1.65x10^{-}2$	0.861	0.909	NLT < NASH-W
cg08008286	*SULT1A1*	0.953	0.467	$5.12x10^{-5}$	0.833	1	NLT < NASH-W
cg08450091	*N/A*	0.953	0.704	$7.64x10^{-11}$	0.889	0.955	NLT > NASH-W
cg08858272	*NALCN*	0.953	0.412	$4.4x10^{-6}$	0.889	0.955	NLT > NASH-W

cg10195415	*TRIM65*	0.953	0.557	2.04×10^{-2}	0.889	0.909	NLT < NASH-W
cg10888281	*CEP68*	0.953	0.322	1.42×10^{-5}	0.778	1	NLT < NASH-W
cg11010178	*C6orf174*	0.953	0.203	1.23×10^{-6}	0.861	0.955	NLT < NASH-W
cg11820154	*N/A*	0.953	0.513	1.70×10^{-5}	0.972	0.909	NLT < NASH-W
cg11822175	*N/A*	0.953	0.692	3.75×10^{-2}	0.944	0.818	NLT > NASH-W
cg11957128	*LOC150786*	0.953	0.607	1.20×10^{-6}	0.806	1	NLT > NASH-W
cg13560760	*PMPCA*	0.953	0.635	7.01×10^{-7}	0.806	1	NLT > NASH-W
cg14072016	*DIP2C*	0.953	0.395	4.09×10^{-2}	0.833	1	NLT < NASH-W
cg15371806	*KIF25*	0.953	0.692	1.37×10^{-4}	0.889	0.955	NLT > NASH-W
cg15945209	*N/A*	0.953	0.594	3.86×10^{-5}	1	0.773	NLT > NASH-W
cg17304646	*N/A*	0.953	0.346	6.11×10^{-4}	0.944	0.864	NLT < NASH-W
cg23932332	*DUSP10*	0.953	0.6	1.52×10^{-3}	0.833	1	NLT < NASH-W
cg24939819	*GPN2*	0.953	0.473	7.25×10^{-5}	0.861	0.955	NLT < NASH-W
cg25270498	*METRNL*	0.953	0.194	9.85×10^{-8}	0.861	0.955	NLT < NASH-W
cg26385097	*N/A*	0.953	0.366	4.22×10^{-6}	0.944	0.864	NLT > NASH-W
cg26414000	*C1orf52*	0.953	0.336	1.73×10^{-3}	0.833	0.955	NLT < NASH-W
cg26583598	*ZSWIM6*	0.953	0.727	9.95×10^{-7}	0.972	0.864	NLT < NASH-W
cg09232069	*SEC14L1*	0.953	0.319	1.27×10^{-3}	0.943	0.864	NLT < NASH-W
cg00334245	*N/A*	0.952	0.136	7.97×10^{-4}	0.861	1	NLT < NASH-W
cg02642549	*LHFPL2*	0.952	0.382	6.44×10^{-8}	0.833	0.955	NLT > NASH-W
cg03674076	*CSNK2A1*	0.952	0.291	3.79×10^{-6}	0.917	0.909	NLT < NASH-W
cg05341610	*ZNF264*	0.952	0.687	2.32×10^{-5}	0.972	0.818	NLT > NASH-W
cg06852575	*ZNF257*	0.952	0.264	3.68×10^{-8}	0.889	0.955	NLT < NASH-W
cg07039975	*DCTN1*	0.952	0.507	7.93×10^{-3}	0.944	0.864	NLT < NASH-W
cg07058086	*KIF13B*	0.952	0.212	1.01×10^{-7}	0.889	0.955	NLT < NASH-W
cg07100957	*ZNF324*	0.952	0.722	3.73×10^{-6}	0.972	0.773	NLT > NASH-W
cg08072980	*IKZF1*	0.952	0.653	8.63×10^{-9}	0.861	1	NLT > NASH-W
cg08914926	*CLIP1*	0.952	0.683	4.82×10^{-6}	0.917	0.909	NLT < NASH-W
cg11354266	*LOC285954*	0.952	0.409	4.12×10^{-2}	0.972	0.864	NLT < NASH-W
cg11527367	*C1orf152*	0.952	0.638	3.23×10^{-9}	0.917	0.909	NLT > NASH-W
cg12728623	*COMT*	0.952	0.467	6.76×10^{-4}	0.917	0.909	NLT < NASH-W
cg12895701	*N/A*	0.952	0.73	3.65×10^{-7}	0.972	0.818	NLT < NASH-W
cg15164048	*ARHGEF10*	0.952	0.749	4.66×10^{-6}	1	0.818	NLT > NASH-W
cg17806401	*LYN*	0.952	0.572	2.14×10^{-5}	0.917	0.909	NLT > NASH-W
cg18440889	*SHANK2*	0.952	0.767	1.00×10^{-8}	0.833	1	NLT > NASH-W
cg18593660	*NAV2*	0.952	0.425	2.18×10^{-6}	0.917	0.909	NLT < NASH-W
cg20023538	*N/A*	0.952	0.639	7.73×10^{-6}	1	0.773	NLT > NASH-W
cg24738257	*N/A*	0.952	0.646	3.42×10^{-6}	0.889	0.955	NLT > NASH-W
cg24958366	*N/A*	0.952	0.459	2.03×10^{-6}	0.944	0.864	NLT > NASH-W
cg25098401	*LCE2B*	0.952	0.687	4.78×10^{-3}	0.861	1	NLT > NASH-W
cg26215406	*MCF2L2*	0.952	0.652	1.70×10^{-3}	0.917	0.909	NLT > NASH-W
cg26520012	*N/A*	0.952	0.288	9.16×10^{-5}	0.917	0.909	NLT < NASH-W
cg13719443	*LOC285847*	0.952	0.413	9.08×10^{-8}	0.943	0.909	NLT > NASH-W
cg14288091	*NIPAL2*	0.952	0.186	7.29×10^{-6}	0.914	0.909	NLT > NASH-W
cg16898066	*HIST1H2BA*	0.952	0.718	3.70×10^{-6}	0.857	0.955	NLT > NASH-W
cg00316520	*N/A*	0.951	0.727	1.40×10^{-5}	0.889	0.909	NLT > NASH-W
cg00318436	*PARD6A*	0.951	0.528	7.33×10^{-7}	0.944	0.909	NLT < NASH-W
cg01554453	*ENPP1*	0.951	0.275	8.65×10^{-8}	0.861	1	NLT < NASH-W
cg02539829	*PGAP1*	0.951	0.866	2.09×10^{-5}	1	0.818	NLT < NASH-W
cg02718464	*SUGT1L1*	0.951	0.806	5.15×10^{-5}	0.861	0.955	NLT > NASH-W
cg03611809	*N/A*	0.951	0.769	1.33×10^{-6}	0.917	0.864	NLT < NASH-W
cg03853620	*PDK4*	0.951	0.566	4.64×10^{-5}	0.861	0.955	NLT < NASH-W
cg04020984	*LOC100270746*	0.951	0.259	9.48×10^{-8}	0.889	1	NLT < NASH-W
cg04396721	*MOSC1*	0.951	0.499	2.73×10^{-2}	0.889	0.864	NLT < NASH-W
cg05064489	*SEC31B*	0.951	0.47	1.10×10^{-4}	0.944	0.909	NLT < NASH-W
cg05234035	*N/A*	0.951	0.245	2.40×10^{-5}	0.944	0.864	NLT < NASH-W
cg05861743	*APOM*	0.951	0.808	1.64×10^{-7}	0.944	0.909	NLT < NASH-W
cg07223240	*N/A*	0.951	0.701	2.90×10^{-7}	0.889	0.909	NLT > NASH-W

cg09212610	*GABARAPL3*	0.951	0.791	2.04×10^{-7}	0.917	0.909	NLT > NASH-W
cg09580859	*ZC3H3*	0.951	0.653	4.45×10^{-5}	0.889	0.909	NLT < NASH-W
cg11586591	*SORL1*	0.951	0.577	4.19×10^{-4}	0.833	0.955	NLT < NASH-W
cg11772086	*MCC*	0.951	0.509	1.14×10^{-3}	0.889	0.909	NLT < NASH-W
cg14579098	*ILF3*	0.951	0.645	2.92×10^{-5}	0.833	0.955	NLT > NASH-W
cg16074383	*ZNF219*	0.951	0.713	3.19×10^{-6}	0.889	0.909	NLT < NASH-W
cg16117273	*PRKD1*	0.951	0.29	2.73×10^{-6}	0.889	0.955	NLT < NASH-W
cg16282910	*N/A*	0.951	0.657	4.96×10^{-8}	0.833	1	NLT > NASH-W
cg16478145	*TFPI*	0.951	0.544	3.53×10^{-4}	0.917	0.909	NLT < NASH-W
cg17461271	*N/A*	0.951	0.695	8.05×10^{-8}	0.917	0.955	NLT > NASH-W
cg18642567	*RPGRIP1*	0.951	0.722	7.52×10^{-8}	0.833	1	NLT > NASH-W
cg18690712	*DNAH12*	0.951	0.645	1.91×10^{-6}	0.944	0.864	NLT > NASH-W
cg19912309	*N/A*	0.951	0.639	2.96×10^{-5}	0.917	0.864	NLT > NASH-W
cg20414217	*HSD3B2*	0.951	0.616	4.64×10^{-8}	0.806	1	NLT > NASH-W

[a]Probe ID of the Infinium HumanMethylation450 BeadChip. [b]National Center for Biotechnology Information (NCBI) database (Genome Build 37). [c]The Youden index was used as a cutoff value for discriminating NASH-W samples from NLT samples for each probe. [d]Sensitivity was defined as the ratio of the number of tissue samples diagnosed as NASH-W based on the criteria relative to the exact number of the NASH-W samples. [e]Specificity was defined as the ratio of the number of tissue samples not diagnosed as NASH-W based on the criteria relative to the exact number of the NLT samples. [f]NASH-W＜NLT: the sample was predicted to be NASH-W and/or carcinogenic risk-positive when its DNA methylation level was lower than the cutoff value; NASH-W>NLT: the sample was predicted to be NASH-W and/or carcinogenic risk-positive when its DNA methylation level was higher than the cutoff value. N/A, not annotated (designed in the intergenic regions).

Additional file 5: Supplementary Table S4 Seventy statistically significant pathway maps in which the 415 probes showing area under the curve (AUC) values more than 0.95 for discrimination of non-cancerous liver tissue samples showing non-alcoholic steatohepatitis from patients with hepatocellular carcinoma (NASH-W) from normal liver tissue (NLT) samples, and were designed for the 339 genes, were significantly (*P* <0.05) accumulated, as revealed by Reactome pathway analysis (https://reactome.org).

ID	Description	*P*	Involved genes showing AUC values of more than 0.95 for discrimination of MASH-W samples from NLT samples
R-HSA-210990	PECAM1 interactions	0.000672	*INPP5D, LYN, SRC*
R-HSA-3247509	Chromatin modifying enzymes	0.000904	*NSD2, SMARCA4, H2AC13, H2BC13, H3C3, KAT6B, RCOR1, ELP5, MRGBP, EHMT1, H2BC1, H2AC1*
R-HSA-4839726	Chromatin organization	0.000904	*NSD2, SMARCA4, H2AC13, H2BC13, H3C3, KAT6B, RCOR1, ELP5, MRGBP, EHMT1, H2BC1, H2AC1*
R-HSA-1632852	Macroautophagy	0.001041	*WIPI2, PARK7, CSNK2A1, SRC, PCNT, TOMM20, PRKAG2, WDR45B*
R-HSA-3214847	HATs acetylate histones	0.001375	*H2AC13, H2BC13, H3C3, KAT6B, ELP5, MRGBP, H2BC1, H2AC1*
R-HSA-9663891	Selective autophagy	0.001375	*PARK7, CSNK2A1, SRC, PCNT, TOMM20, PRKAG2*
R-HSA-68886	M Phase	0.001629	*PPP2R2D, CKAP5, DCTN1, RCC2, CTDNEP1, CSNK2A1, CLIP1, UBE2I, H2BC13, H3C3, PCNT, RAN, GORASP2, H2BC1, TUBB*
R-HSA-9612973	Autophagy	0.002030	*WIPI2, PARK7, CSNK2A1, SRC, PCNT, TOMM20, PRKAG2, WDR45B*
R-HSA-3214815	HDACs deacetylate histones	0.002940	*H2AC13, H2BC13, H3C3, RCOR1, H2BC1, H2AC1*
R-HSA-9620244	Long-term potentiation	0.005412	*GRIN2A, DLG2, SRC*
R-HSA-5693565	Recruitment and ATM-mediated phosphorylation of repair and signaling proteins at DNA double strand breaks	0.006073	*NSD2, UBE2I, H2BC13, BRCA1, H2BC1*
R-HSA-5693606	DNA Double Strand Break Response	0.006411	*NSD2, UBE2I, H2BC13, BRCA1, H2BC1*
R-HSA-373752	Netrin-1 signaling	0.006715	*NEO1, SRC, PITPNA, MYO10*
R-HSA-1221632	Meiotic synapsis	0.006763	*ACD, UBE2I, H2BC13, BRCA1, H2BC1*
R-HSA-2559582	Senescence-Associated Secretory Phenotype (SASP)	0.006925	*RPS6KA2, CDK6, H2BC13, H3C3, EHMT1, H2BC1*
R-HSA-438064	Post NMDA receptor activation events	0.007128	*GRIN2A, RPS6KA2, DLG2, SRC, PRKAG2*
R-HSA-1483191	Synthesis of PC	0.008382	*PHOSPHO1, CSNK2A1, SLC44A4*
R-HSA-1500620	Meiosis	0.008863	*ACD, UBE2I, H2BC13, H3C3, BRCA1, H2BC1*
R-HSA-5205647	Mitophagy	0.009247	*CSNK2A1, SRC, TOMM20*
R-HSA-8934903	Receptor Mediated Mitophagy	0.011386	*CSNK2A1, SRC*
R-HSA-442755	Activation of NMDA receptors and postsynaptic events	0.013191	*GRIN2A, RPS6KA2, DLG2, SRC, PRKAG2*
R-HSA-112308	Presynaptic depolarization and calcium channel opening	0.013528	*CACNB3, CACNG2*
R-HSA-2029481	FCGR activation	0.013528	*LYN, SRC*
R-HSA-5693567	HDR through Homologous Recombination (HRR) or Single Strand Annealing (SSA)	0.014844	*NSD2, RAD51B, UBE2I, H2BC13, BRCA1, H2BC1*
R-HSA-1268020	Mitochondrial protein import	0.015756	*CHCHD7, TOMM20, PMPCA, LDHD*
R-HSA-5693607	Processing of DNA double-strand break ends	0.016240	*NSD2, UBE2I, H2BC13, BRCA1, H2BC1*
R-HSA-5689901	Metalloprotease DUBs	0.018015	*H2AC13, BRCA1, H2AC1*
R-HSA-5693538	Homology Directed Repair	0.018108	*NSD2, RAD51B, UBE2I, H2BC13, BRCA1, H2BC1*
R-HSA-5693571	Nonhomologous End-Joining (NHEJ)	0.020240	*NSD2, H2BC13, BRCA1, H2BC1*
R-HSA-68875	Mitotic Prophase	0.020539	*PPP2R2D, CTDNEP1, H2BC13, H3C3, GORASP2, H2BC1*
R-HSA-354194	GRB2:SOS provides linkage to MAPK signaling for Integrins	0.020895	*RAP1B, SRC*
R-HSA-372708	p130Cas linkage to MAPK signaling for integrins	0.020895	*RAP1B, SRC*
R-HSA-3772470	Negative regulation of TCF-dependent signaling by WNT ligand antagonists	0.020895	*LRP5, DKK1*
R-HSA-1474165	Reproduction	0.021180	*ACD, UBE2I, H2BC13, H3C3, BRCA1, H2BC1*
R-HSA-112315	Transmission across Chemical Synapses	0.021203	*CACNB3, COMT, GRIN2A, RPS6KA2, DLG2, GABBR1, SRC, CACNG2, PRKAG2*
R-HSA-380259	Loss of Nlp from mitotic centrosomes	0.021221	*CKAP5, DCTN1, PCNT, TUBB*
R-HSA-380284	Loss of proteins required for interphase microtubule organization from the centrosome	0.021221	*CKAP5, DCTN1, PCNT, TUBB*
R-HSA-1433559	Regulation of KIT signaling	0.023647	*LYN, SRC*
R-HSA-2730905	Role of LAT2/NTAL/LAB on calcium mobilization	0.023647	*LYN, LAT2*
R-HSA-8854518	AURKA Activation by TPX2	0.024338	*CKAP5, DCTN1, PCNT, TUBB*
R-HSA-1912422	Pre-NOTCH Expression and Processing	0.024516	*RFNG, H2BC13, H3C3, MAML3, H2BC1*
R-HSA-8934593	Regulation of RUNX1 Expression and Activity	0.026538	*CDK6, SRC*
R-HSA-157118	Signaling by NOTCH	0.026624	*RFNG, H2BC13, H3C3, SMAD3, HDAC4, IKZF1, MAML3, H2BC1*
R-HSA-1839117	Signaling by cytosolic FGFR1 fusion mutants	0.029563	*LRRFIP1, CUX1*
R-HSA-2559583	Cellular Senescence	0.029720	*RPS6KA2, ACD, CDK6, H2BC13, H3C3, EHMT1, H2BC1*
R-HSA-5663202	Diseases of signal transduction by growth factor receptors and second messengers	0.030938	*RAP1B, LYN, DCTN1, LRRFIP1, CUX1, LRP5, SRC, SMAD3, HDAC4, DUSP10, DKK1, MAML3*
R-HSA-9610379	HCMV Late Events	0.030981	*H2AC13, H2BC13, H3C3, H2BC1, H2AC1*
R-HSA-3214858	RMTs methylate histone arginines	0.031367	*SMARCA4, H2AC13, H3C3, H2AC1*
R-HSA-69231	Cyclin D associated events in G1	0.033724	*LYN, CDK6, SRC*
R-HSA-69236	G1 Phase	0.033724	*LYN, CDK6, SRC*
R-HSA-68877	Mitotic Prometaphase	0.034953	*CKAP5, DCTN1, RCC2, CSNK2A1, CLIP1, PCNT, TUBB*
R-HSA-380270	Recruitment of mitotic centrosome proteins and complexes	0.035284	*CKAP5, DCTN1, PCNT, TUBB*
R-HSA-380287	Centrosome maturation	0.035284	*CKAP5, DCTN1, PCNT, TUBB*
R-HSA-112314	Neurotransmitter receptors and postsynaptic signal transmission	0.035748	*GRIN2A, RPS6KA2, DLG2, GABBR1, SRC, CACNG2, PRKAG2*
R-HSA-9013695	NOTCH4 Intracellular Domain Regulates Transcription	0.036001	*SMAD3, MAML3*
R-HSA-9669938	Signaling by KIT in disease	0.036001	*LYN, SRC*
R-HSA-9670439	Signaling by phosphorylated juxtamembrane, extracellular and kinase domain KIT mutants	0.036001	*LYN, SRC*
R-HSA-5688426	Deubiquitination	0.036707	*TNIP2, H2AC13, H2BC13, SMAD3, BRCA1, TOMM20, INO80, H2BC1, H2AC1*
R-HSA-389513	CTLA4 inhibitory signaling	0.039404	*LYN, SRC*
R-HSA-5620916	VxPx cargo-targeting to cilium	0.039404	*ARF4, EXOC2*
R-HSA-9008059	Interleukin-37 signaling	0.039404	*PTPN9, SMAD3*
R-HSA-5693532	DNA Double-Strand Break Repair	0.040819	*NSD2, RAD51B, UBE2I, H2BC13, BRCA1, H2BC1*
R-HSA-912446	Meiotic recombination	0.040930	*H2BC13, H3C3, BRCA1, H2BC1*
R-HSA-438066	Unblocking of NMDA receptors, glutamate binding and activation	0.042923	*GRIN2A, DLG2*
R-HSA-9613829	Chaperone Mediated Autophagy	0.042923	*PARK7, PCNT*

R-HSA-9617324	Negative regulation of NMDA receptor-mediated neuronal transmission	0.042923	*GRIN2A, DLG2*
R-HSA-171306	Packaging Of Telomere Ends	0.043546	*ACD, H2BC13, H2BC1*
R-HSA-2565942	Regulation of PLK1 Activity at G2/M Transition	0.043934	*CKAP5, DCTN1, PCNT, TUBB*
R-HSA-449147	Signaling by Interleukins	0.046945	*RPS6KA2, RAP1B, INPP5D, LYN, SMARCA4, TNIP2, PTPN9, H3C3, SMAD3, PITPNA, IL20, TOLLIP*
R-HSA-201722	Formation of the beta-catenin:TCF transactivating complex	0.048667	*SMARCA4, H2BC13, H3C3, H2BC1*

Additional file 6: Supplementary Table S5 Correlation between levels of DNA methylation and expression for the 13 genes included in Table 2 and for which expression data are available in the The Cancer Genome Atlas database (https://www.cancer.gov/about-nci/organization/ccg/research/structural-genomics/tcga).

Probe ID	Gene symbol	*P*	Correlation coefficient (r)
cg05861743	*APOM**	1.35×10^{-29}	-0.533
cg19861632	*GPR125**	1.19×10^{-8}	-0.286
cg14950303	*FOXD4L1**	2.33×10^{-5}	0.214
cg09580859	*ZC3H3*	7.68×10^{-4}	0.171
cg14288091	*NIPAL2*	5.19×10^{-3}	-0.142
cg13719443	*LOC285847*	8.21×10^{-3}	-0.135
cg26894854	*SLC45A3*	4.52×10^{-2}	-0.102
cg23785719	*STK17A*	0.12	-0.079
cg26583598	*ZSWIM6*	0.269	-0.057
cg05393736	*NRBF2*	0.354	0.047
cg18210511	*ZC3H3*	0.664	0.022
cg22083325	*LOC440461*	0.687	-0.021
cg00431813	*C7orf50*	0.749	0.016

*Genes showing *P* values of less than 0.05 and r values of less than -0.2 or more than 0.2.

Additional file 7: Supplementary Table S6 Comparison of DNA methylation levels based on the Infinium assay between samples of normal healthy liver tissue (GSE107038) deposited in the GEO database (https://www.ncbi.nlm.nih.gov/geo/query/acc.cgi?acc=GSE107038) and non-cancerous liver tissue samples showing non-alcoholic steatohepatitis from our cohort of patients with hepatocellular carcinoma (NASH-W).

Probe ID	GSE107038 (mean± standard deviation [SD])			NASH-W (mean±SD)			$\Delta\beta_{NASH\text{-}W\text{-}GSE107038}$	*P* (Welch's *t* test)
cg00431813	0.452	±	0.069	0.629	±	0.085	0.177	5.23×10^{-10}
cg04662828	0.682	±	0.073	0.506	±	0.053	-0.176	2.27×10^{-15}
cg05393736	0.675	±	0.049	0.749	±	0.039	0.074	3.00×10^{-8}
cg05861743	0.717	±	0.065	0.843	±	0.049	0.126	1.46×10^{-11}
cg08912801	0.647	±	0.065	0.531	±	0.07	-0.116	1.03×10^{-7}
cg09580822	0.837	±	0.076	0.599	±	0.109	-0.239	1.90×10^{-10}
cg09580859	0.609	±	0.119	0.784	±	0.113	0.175	7.53×10^{-7}
cg11152384	0.538	±	0.09	0.36	±	0.083	-0.178	4.69×10^{-10}
cg11820154	0.456	±	0.056	0.546	±	0.054	0.09	1.98×10^{-7}
cg13719443	0.462	±	0.082	0.35	±	0.063	-0.112	1.79×10^{-7}
cg14288091	0.279	±	0.075	0.146	±	0.049	-0.133	1.33×10^{-11}
cg14950303	0.489	±	0.043	0.583	±	0.045	0.094	6.36×10^{-10}
cg15050398	0.752	±	0.039	0.479	±	0.058	-0.273	1.61×10^{-19}
cg16319310	0.808	±	0.064	0.587	±	0.027	-0.221	3.33×10^{-26}
cg18210511	0.464	±	0.076	0.603	±	0.078	0.14	2.68×10^{-8}
cg19861632	0.512	±	0.058	0.605	±	0.056	0.093	2.14×10^{-7}
cg22083325	0.163	±	0.056	0.289	±	0.052	0.125	2.16×10^{-11}
cg23785719	0.133	±	0.028	0.319	±	0.043	0.186	2.36×10^{-18}
cg26385097	0.504	±	0.116	0.293	±	0.105	-0.211	2.85×10^{-9}
cg26583598	0.642	±	0.097	0.776	±	0.087	0.134	1.12×10^{-6}
cg26894854	0.464	±	0.051	0.54	±	0.052	0.075	2.07×10^{-6}

Additional file 8: Supplementary Table S7 Multivariate analysis using the 91 samples of non-cancerous liver tissue showing non-alcoholic steatohepatitis (NASH) derived from biopsy specimens of patients with morbid obesity but without hepatocellular carcinoma (HCC) (NASH-O samples) and the 22 samples of non-cancerous liver tissue showing NASH derived from partial hepatectomy specimens of patients with HCC (NASH-W samples) in the initial cohorts.

Probe ID[a]	Independent variable	Odds ratio[b]	95% confidence interval	*P* value[c]
cg00431813	Ballooning[d]	5.786	0.132-252.827	0.362
	Brunt classification[e]	0.686	0.275-1.714	0.420
	DNA methylation quantification	232.059	31.166-1727.915	1.05x10^{-7}
cg05393736	Ballooning[d]	4.286	0.269-68.340	0.303
	Brunt classification[c]	0.972	0.472-2.001	0.938
	DNA methylation quantification	44.977	10.934-185.009	1.33x10^{-7}
cg09580822	Ballooning[d]	1.378	0.124-15.383	0.795
	Brunt classification[e]	0.553	0.248-1.235	0.148
	DNA methylation quantification	180.610	23.957-1361.603	4.61x10^{-7}
cg11820154	Ballooning[d]	0.460	0.031-6.765	0.571
	Brunt classification[c]	0.347	0.100-1.207	0.096
	DNA methylation quantification	1138.492	52.740-24576.521	7.13x10^{-6}
cg14950303	Ballooning[d]	1.256	0.114-13.824	0.853
	Brunt classification[c]	0.702	0.315-1.565	0.387
	DNA methylation quantification	144.050	21.556- 962.621	2.92x10^{-7}
cg15050398	Ballooning[d]	7.485	0.656-85.377	0.105
	Brunt classification[e]	1.772	0.873-3.600	0.113
	DNA methylation quantification	39.410	10.225-151.901	9.44x10^{-8}
cg23785719	Ballooning[d]	2.288	0.048-109.285	0.675
	Brunt classification[e]	2.717	0.959-7.697	0.060
	DNA methylation quantification	245.432	30.392-1981.975	2.42x10^{-7}
cg26583598	Ballooning[d]	0.692	0.051-9.460	0.782
	Brunt classification[e]	0.286	0.090-0.915	0.035
	DNA methylation quantification	1035.707	54.365-19731.273	3.89x10^{-6}
cg18210511	Ballooning[d]	8.990	0.219-369.308	0.247
	Brunt classification[e]	0.482	0.207-1.123	0.091
	DNA methylation quantification	248.725	30.517-2027.198	2.56x10^{-7}
cg05861743	Ballooning[d]	1.491	0.133-16.687	0.746
	Brunt classification[e]	0.503	0.229-1.106	0.088
	DNA methylation quantification	190.885	24.574-1482.750	5.14x10^{-7}
cg14288091	Ballooning[d]	1.606	0.152-16.949	0.694
	Brunt classification[e]	0.759	0.344-1.675	0.494

	DNA methylation quantification	99.726	16.280-610.913	6.46x10^{-7}
cg26385097	Ballooning[d]	1.577	0.145-17.140	0.708
	Brunt classification[e]	0.595	0.275-1.288	0.188
	DNA methylation quantification	141.119	20.394-976.513	5.30x10^{-7}
cg22083325	Ballooning[d]	6.795	0.307-150.345	0.225
	Brunt classification[e]	0.632	0.286-1.397	0.257
	DNA methylation quantification	90.209	16.093-505.661	3.07x10^{-7}
cg09580859	Ballooning[d]	9.892	0.299-327.815	0.200
	Brunt classification[e]	0.506	0.226-1.133	0.098
	DNA methylation quantification	180.393	24.567-1324.612	3.27x10^{-7}
cg13719443	Ballooning[d]	1.774	0.166-18.957	0.635
	Brunt classification[e]	0.609	0.278-1.335	0.216
	DNA methylation quantification	120.359	17.878-810.272	8.48x10^{-7}
cg26894854	Ballooning[d]	1.782	0.169-18.816	0.631
	Brunt classification[e]	0.586	0.273-1.261	0.172
	DNA methylation quantification	83.537	14.260-489.391	9.28x10^{-7}
cg04662828	Ballooning[d]	14.655	1.172-183.220	0.037
	Brunt classification[e]	1.036	0.544-1.973	0.915
	DNA methylation quantification	35.014	8.406-145.841	1.04x10^{-6}
cg11152384	Ballooning[d]	1.865	0.172-20.186	0.608
	Brunt classification[e]	0.528	0.248-1.125	0.098
	DNA methylation quantification	140.210	19.811-992.306	7.38x10^{-7}
cg08912801	Ballooning[d]	9.581	0.420-218.813	0.157
	Brunt classification[e]	0.662	0.324-1.352	0.258
	DNA methylation quantification	91.389	15.135-551.828	8.58x10^{-7}
cg16319310	Ballooning[d]	4.220	0.268-66.499	0.306
	Brunt classification[e]	2.148	0.963-4.788	0.062
	DNA methylation quantification	70.412	12.597-393.575	1.26x10^{-6}
cg19861632	Ballooning[d]	2.047	0.191-21.929	0.554
	Brunt classification[e]	0.610	0.302-1.232	0.168
	DNA methylation quantification	207.072	20.883-2053.237	5.20x10^{-6}

[a]Probe ID of the Infinium HumanMethylation450 BeadChip. [b]Top 10 CpG sites showing the highest odds ratio of being NASH-W samples are summarized in Table 3. [c]*P* values less than 0.05 are underlined.[d]Ballooning was evaluated according to the non-alcoholic fatty disease (NAFLD) activity score (NAS) (Ref. 26) [e]Fibrosis stage was defined according to the Brunt classification (Ref. 27).

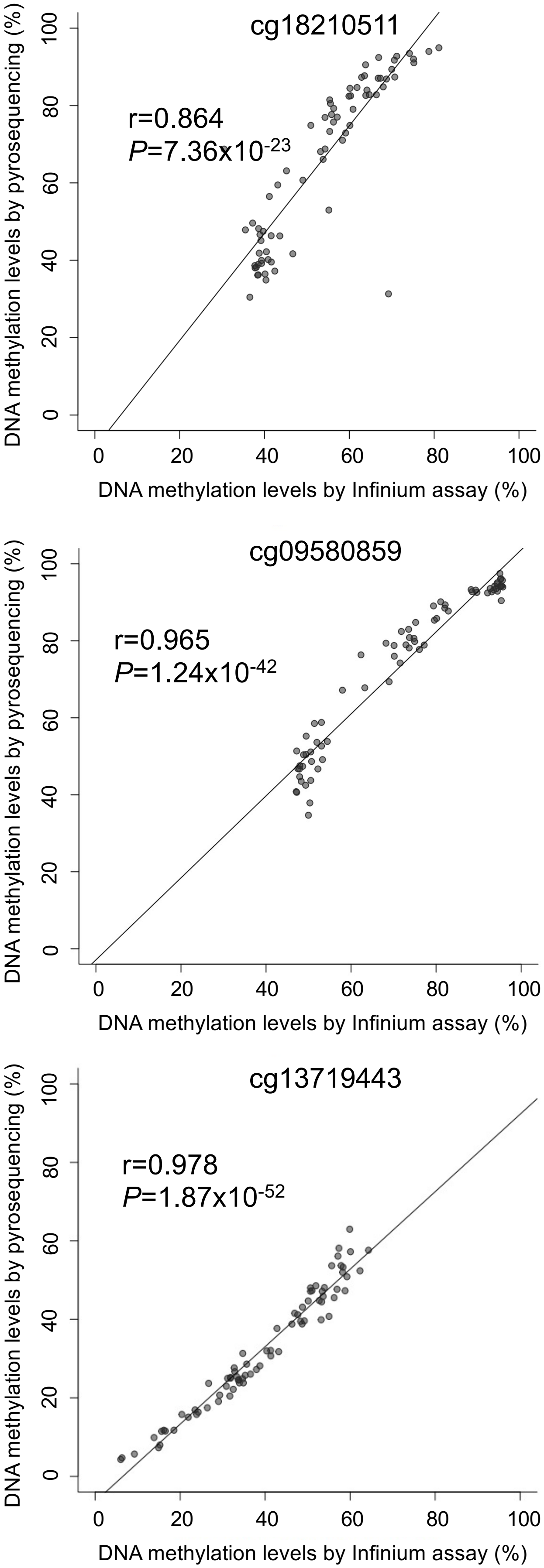

Additional file 9: Supplementary Figure S2 Correlation between DNA methylation levels based on the Infinium assay and such levels based on pyrosequencing using samples of normal liver tissue (n=34), non-cancerous liver tissue showing non-alcoholic steatohepatitis from partial hepatectomy specimens from patients with hepatocellular carcinoma (n=22), and cancerous tissue samples (n = 22) in the initial cohort. Infinium data were successfully verified by pyrosequencing. (P<7.36$\times$10^{-23} and Pearson correlation coefficient [r]>0.864).

Additional file 10: Supplementary Table S8 Correlation between DNA methylation levels based on the Infinium assay of the 21 marker CpG sites included in Table 2 and obesity in patients with nonalcoholic steatohepatitis (NASH) with and without hepatoceullar carcinoma in the initial and validation cohorts.

Probe ID	DNA methylation levels based on Infinium assay (mean±standard deviation)		
	Non-obese NASH paients (body mass index [BMI]<25) (n=17)	Obese NASH paients (BMI≧25) (n=22)	*P* (Welch's *t* test)
cg00431813	0.591 ± 0.106	0.614 ± 0.109	0.519
cg04662828	0.512 ± 0.045	0.503 ± 0.041	0.522
cg05393736	0.725 ± 0.037	0.708 ± 0.056	0.240
cg05861743	0.815 ± 0.061	0.831 ± 0.055	0.403
cg08912801	0.560 ± 0.078	0.528 ± 0.102	0.276
cg09580822	0.617 ± 0.106	0.613 ± 0.131	0.923
cg09580859	0.749 ± 0.145	0.750 ± 0.138	0.974
cg11152384	0.399 ± 0.099	0.359 ± 0.109	0.237
cg11820154	0.524 ± 0.067	0.528 ± 0.063	0.877
cg13719443	0.374 ± 0.096	0.376 ± 0.11	0.950
cg14288091	0.178 ± 0.071	0.160 ± 0.075	0.463
cg14950303	0.560 ± 0.048	0.577 ± 0.052	0.302
cg15050398	0.460 ± 0.043	0.462 ± 0.052	0.886
cg16319310	0.579 ± 0.019	0.575 ± 0.041	0.670
cg18210511	0.583 ± 0.104	0.579 ± 0.088	0.901
cg19861632	0.587 ± 0.045	0.597 ± 0.061	0.560
cg22083325	0.266 ± 0.042	0.282 ± 0.056	0.304
cg23785719	0.313 ± 0.037	0.282 ± 0.041	0.016
cg26385097	0.332 ± 0.124	0.298 ± 0.136	0.411
cg26583598	0.729 ± 0.107	0.727 ± 0.111	0.939
cg26894854	0.525 ± 0.046	0.529 ± 0.073	0.854

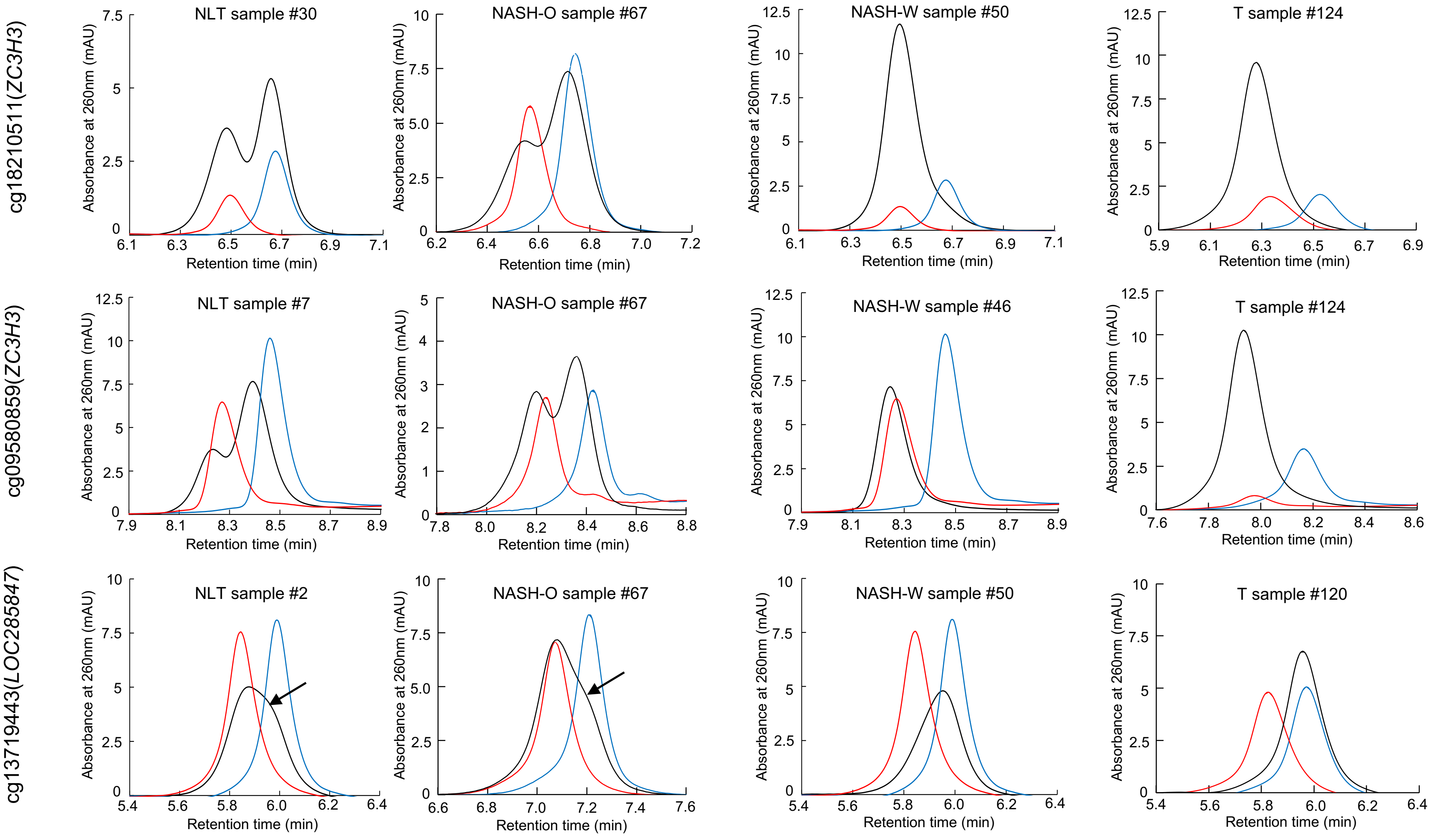

Additional file 11: Supplementary Figure S3 Representative chromatograms obtained by high-performance liquid chromatography analysis using a newly developed anion-exchange column for marker CpG sites in specimens of normal liver tissue (NLT), non-cancerous liver tissue (N) showing non-alcoholic steatohepatitis (NASH) derived from partial hepatectomy specimens from patients without hepatocellular carcinoma (HCC) (NASH-O), N samples showing NASH from patients with HCC (NASH-W), and cancerous tissue (T) samples. The chromatogram patterns (bimodal peak or single peak with a shoulder [indicated by an arrow]) for most of the NASH-O samples are similar to those for NLT samples, whereas those for T samples (single peak) are similar to those for NASH-W samples.

Additional file 12: Supplementary Table S9 Correlation between relative methylation rates based on high-performance liquid chromatography (HPLC) and the sex and age of nonalcoholic steatohepatitis (NASH) patients with and without hepatoceullar carcinoma in the initial and validation cohorts from whom HLPC data had been obtained (n=34).

Probe ID	Correlation with sex			Correlation with age	
	Relative methylation rates (mean±standard deviation)		*P* (Welch's *t* test)	*P*	Correlation coefficient (r)
	Male (n=29)	Female (n=5)			
cg18210511	83.6 ± 20.3	100.4 ± 14.1	0.0540	0.0994	0.287
cg09580859	96.1 ± 17.2	108.7 ± 11.1	0.0663	0.0345	0.364
cg13719443	32.4 ± 10.0	30.6 ± 9.1	0.7147	0.6018	-0.093
